# GEI-8, a Homologue of Vertebrate Nuclear Receptor Corepressor NCoR/SMRT, Regulates Gonad Development and Neuronal Functions in *Caenorhabditis elegans*

**DOI:** 10.1371/journal.pone.0058462

**Published:** 2013-03-06

**Authors:** Pavol Mikoláš, Johana Kollárová, Kateřina Šebková, Vladimír Saudek, Petr Yilma, Markéta Kostrouchová, Michael W. Krause, Zdenek Kostrouch, Marta Kostrouchová

**Affiliations:** 1 Laboratory of Molecular Biology and Genetics, Institute of Cellular Biology and Pathology, First Faculty of Medicine, Charles University in Prague, Prague, Czech Republic; 2 Laboratory of Molecular Pathology, Institute of Cellular Biology and Pathology, First Faculty of Medicine, Charles University in Prague, Prague, Czech Republic; 3 Laboratory of Molecular Biology, National Institute of Diabetes and Digestive and Kidney Diseases, National Institutes of Health, Bethesda, Maryland, United States of America; Ecole Normale Supérieure de Lyon, France

## Abstract

NCoR and SMRT are two paralogous vertebrate proteins that function as corepressors with unliganded nuclear receptors. Although *C. elegans* has a large number of nuclear receptors, orthologues of the corepressors NCoR and SMRT have not unambiguously been identified in *Drosophila* or *C. elegans*. Here, we identify GEI-8 as the closest homologue of NCoR and SMRT in *C. elegans* and demonstrate that GEI-8 is expressed as at least two isoforms throughout development in multiple tissues, including neurons, muscle and intestinal cells. We demonstrate that a homozygous deletion within the *gei-8* coding region, which is predicted to encode a truncated protein lacking the predicted NR domain, results in severe mutant phenotypes with developmental defects, slow movement and growth, arrested gonadogenesis and defects in cholinergic neurotransmission. Whole genome expression analysis by microarrays identified sets of de-regulated genes consistent with both the observed mutant phenotypes and a role of GEI-8 in regulating transcription. Interestingly, the upregulated transcripts included a predicted mitochondrial sulfide:quinine reductase encoded by Y9C9A.16. This locus also contains non-coding, 21-U RNAs of the piRNA class. Inhibition of the expression of the region coding for 21-U RNAs leads to irregular gonadogenesis in the homozygous *gei-8* mutants, but not in an otherwise wild-type background, suggesting that GEI-8 may function in concert with the 21-U RNAs to regulate gonadogenesis. Our results confirm that GEI-8 is the orthologue of the vertebrate NCoR/SMRT corepressors and demonstrate important roles for this putative transcriptional corepressor in development and neuronal function.

## Introduction

NCoR and SMRT are paralogous vertebrate proteins that were first identified as transcriptional corepressors interacting with unliganded thyroid and retinoid receptors [1], [2]. Both NCoR (a.k.a. NCoR1, NCOR1) and SMRT (a.k.a. NCoR2, NCOR2) knockouts in mice are embryonic lethal suggesting that their regulatory roles are indispensable for normal development [3]. NCoR/SMRT function occurs through the assembly of a repressor complex composed of nuclear hormone receptors (NHRs), histone deactylases (HDACs), and other components [4]. Chromatin remodeling depends on the formation of a stoichiometric complex between SMRT/NCoR and HDAC3 that is mediated by two SANT (a.k.a. MYB) domains located at the N-terminus of NCoR/SMRT. Such domains are present in many nuclear receptor corepressors and related proteins and consist of three alpha-helices folded around a core of three hydrophobic amino acids, which determines its characteristic spatial structure [5]–[7]. The N-terminus proximal SANT1 domain activates the HDAC3 deacetylase [8], [9] and is referred to as the deacetylase activation domain (DAD). A prominent feature of all DAD domains is the absolutely conserved lysine residue (K449 in human SMRT) that promotes HDAC3 activation but not its binding to the complex. The second SANT domain, SANT2, binds unacetylated histone H4 and increases affinity of NCoR/SMRT to HDAC3, suggesting a role for this motif in stabilizing the deacetylated histone tail and blocking its subsequent acetylation [7], [8]. While the SANT2 domain in NCoR/SMRT possesses all of the typical features of a general SANT domain, the presence and structure of the SANT1 domain is unique to NCoR/SMRT and its orthologues [10]. The SANT1 domain contains a characteristic irregular N-terminal helix that is important for forming an additional surface hydrophobic groove that contributes to the interaction with HDAC3. Thus, there are multiple diagnostic domains and amino acid residues that can distinguish NCoR/SMRT orthologues from more general SANT domain-containing proteins.

Although homologues of NCoR/SMRT can be easily identified across vertebrate species, obvious homologues of these corepressors were difficult to identify by sequence homology in either *Drosophila* or *C. elegans*. This is surprising in light of the identification of clear sequence homologues for other NCoR/SMRT corepressor complex components in flies and worms such as the histone deacetylase complex associated factors NuRD and SIN3 [11], [12]. We have taken a bioinformatics approach focusing on the unique features of NCoR/SMRT to identify GEI-8 as a possible NCoR/SMRT homologue in *C. elegans*; Yamamato and colleagues came to the same conclusion while this work was in progress [13]. GEI-8 was originally identified as a GEX-3 binding protein based on yeast-two-hybrid assays [14]; no RNAi phenotypes or functions for GEI-8 have previously been described. We have analyzed the expression of *gei-8* and studied its function using a putative null allele with a large deletion in the *gei-8* coding sequence resulting in a truncated protein product due to a novel stop codon; this truncated product lacks the domain involved in binding of nuclear receptors (NR domain, a.k.a CoRNR box [15]). Our mutant studies demonstrate a role for GEI-8 in development and suggest it is specifically required for germline development and proper cholinergic regulation. Our whole genome expression analysis demonstrates that GEI-8 is required for transcriptional regulation, consistent with its function and orthology to mammalian NCoR/SMRT corepressors.

## Results

### Sequence Analysis

**Figure 1:**
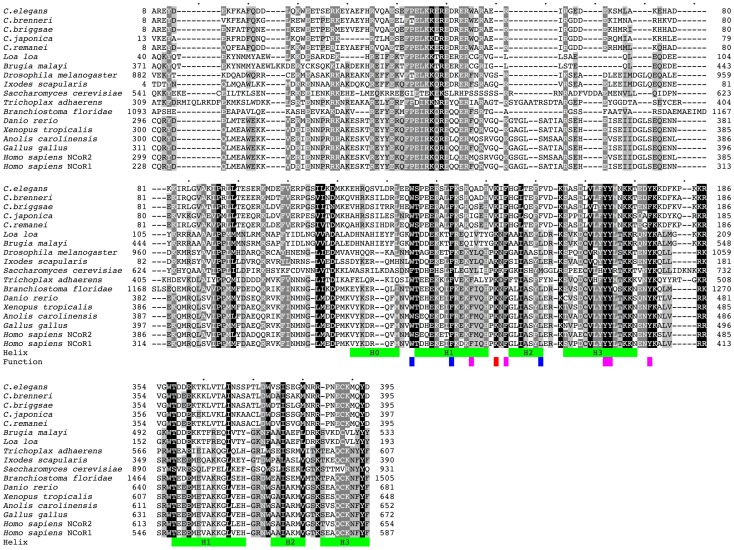
Comparison of N-terminal regions of GEI-8-related proteins to NCoR/SMRT. Sequence alignment of GEI-8 nematode orthologues with their nearest Metazoa/Fungi homologues, both human orthologues NCoR1 and SMRT (NCoR2) are shown. Green bars indicate the position of the alpha-helices in the structure of the upstream DAD domain of human SMRT and homology predicted positions in the second SANT domain. Residues indispensable for regulating HDAC interactions and function are highlighted in blue (needed for the structural integrity), magenta (interaction with HDAC) and red (activation of HDAC). Only the N-terminal part of the sequences is shown. The identical and similar residues are highlighted by different intensity of shading. Sequence identifiers: *C. elegans*: GEI8_CAEEL, *C. brenneri*: CN15693, *C. briggsae*: A8X8F0_CAEBR, *C. remanei*: RP40355, *C. japonica*: JA23925 ABLE03010463.1 ABLE03032768.1 ABLE03032771.1 ABLE03032769.1 ABLE03032772.1, *Loa loa*: E1FVE0_LOALO, *Brugia malayi*: A8NSC3_BRUMA, *Ixodes scapularis*: B7PZ26_IXOSC, *Saccharomyces cerevisiae*: SNT1_YEAST, *Drosophila melanogaster*: Q9VYK0_DROME, *Trichoplax adhaerens*: B3SAN1_TRIAD, *Branchiostoma floridae*: C3XV35_BRAFL, *Danio rerio*: A8B6H7_DANRE, *Xenopus tropicalis*: NCOR1_XENTR AAMC01044136.1, *Anolis carolinensis*: ANOCA15679 2 ENSACAP00000014806; ENSACAT00000015107, *Gallus gallus*: UPI0000E813A6, *Homo sapiens*: NCOR2_HUMAN (NCoR2), *Homo sapiens*: NCOR1_HUMAN (NCoR1). PDB structure 1XC5 was used to determine the position of the helices.

In an effort to identify homologues of NCoR/SMRT in the *C. elegans* proteome, we performed BLAST and PSI-BLAST searches in multiple protein databases [16], [17]. Searches with human NCoR and SMRT sequences returned the sequence annotated as GEI-8 (UniProt GEI8_CAEEL, E value 2e-10), as the best hit. In the reciprocal BLAST, NCoR and SMRT appeared likewise as the best hits for GEI-8 within the human proteome. Although only a small fraction of the entire protein sequence (∼7%) was retrieved by Blast searches, nearly complete protein sequences were recovered in PSI-BLAST after the third iteration. Six GEI-8-related proteins from other Nematoda species (*C. elegans, C. brenneri, C. briggsae, C. remanei, C. japonica, Loa loa* and *Brugia malayi*) were aligned and submitted as a query in PSI-BLAST (**Figure 1**). Sequences were extracted from databases UniProt, Wormbase and Ensembl. Entries for *C. japonica* and *X. tropicalis* were corrected according to NCBI nucleotide sequences using the GeneWise program [18]. An alignment of these nematode GEI-8-related proteins with human NCoR was obtained in the second iteration.

**Figure 2:**
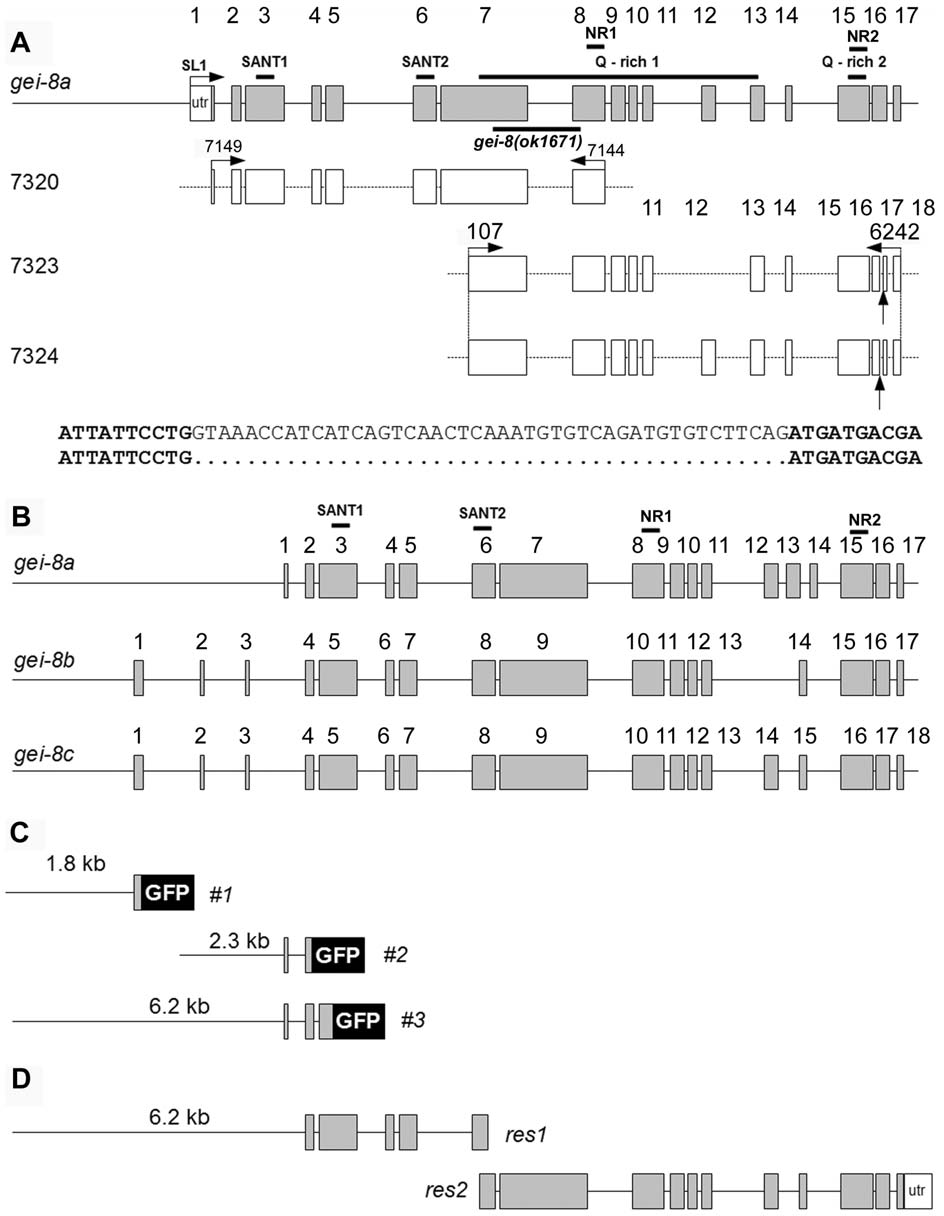
Expression analysis of *gei-8*. (**A**) Schematic representation of the predicted *gei-8a* isoform consisting of 17 exons compared with detected expression. cDNA clones 7320, 7323 and 7324 are indicated with their exons (open rectangles). Expression of exon 12 and 16 is not constant. Exon 12 in cDNA clone 7323 and 45 bp from exon 16 in cDNAs 7323 and 7324 were removed by alternative splicing (bottom two lines). The location of predicted SANT and glutamine-rich interaction domains is marked by lines above the *gei-8a* diagram. Location of *gei-8(ok1671)* mutation used in expression analysis is marked by a line below the *gei-8a* diagram. Two regions identified as putative CoRNR nuclear receptor binding motifs are indicated in the exon 8 and 15 (NR). (**B**) Schematic representation of predicted *gei-8* isoforms *a*, *b* and *c*. (**C**) GFP reporter gene constructs #1, #2 and #3 used for expression analysis. (**D**) Overlapping regions of *gei-8* gDNA used for rescue. The size of the overlapping region is 191 bp.

Multiple sequence alignments resulting from PSI-BLAST were further improved using the profile-to-profile alignment method (PSI-Coffee) [19], however, its quality remained ambiguous in several regions across the protein. All NCoR homologues contain long stretches of low complexity (e.g. 23% of amino acids in GEI-8 or 13% in human NCoR1) that are variable in length. The well conserved N-terminal region from representative *Metazoa/Fungi* NCoR/SMRT is shown in **Figure 1**. The sequence conservation in the C-terminal domains is much lower; all sequences contain many insertions, deletions, prolines, serines and oligoGlu residues that vary between species. This C-terminal variability is evident even within the alignment of the GEI-8-related proteins from the phylogenetically related *Caenorhabditis* species. We also used ClustalW2.0 for identification of putative interaction motifs near the C-terminus. NCoR and SMRT bind nuclear hormone receptors by NR-binding domains consisting of three and two CoRNR-box sequences respectively. The CoRNR-box sequence was previously defined as L.x.x.x.I.x.x.x.I/L [20]; I/L.x.x.I/V.I [21]; L/V.x.x.I/V.I [22]. We identified two putative CoRNR-box like sequences in GEI-8 (**Figure 2A**). The predicted GEI-8 sequence also contains two glutamine rich regions [23] that also might serve as interaction domains.

The most conserved N-terminal regions of the GEI-8 related sequences contain both the DAD and SANT domains with their location and the positions of the conserved helices shown in **Figure 1**. We noted that GEI-8 and related sequences preserve all features known to be essential for correct functioning of NCoR/SMRT as an HDAC-dependent transcriptional corepressor [10] (highlighted in **Figure 1**). These include the number of helices, their topology, the conserved amino acids needed for the integrity of the structure and for the interaction with HDAC and, most importantly, the K159 residue in the loop between helices H1 and H2 that is indispensable for the activation of HDAC3. The helix H0, known to be very irregular in human SMRT, is probably also present although it contains a two amino acid insertion between the second and third helical turn. Based on the sequence analysis, we concluded that GEI-8 bears all major features identified in other NCoR/SMRT orthologues in annotated genomes from other species and is the NR corepressor and NCoR/SMRT orthologue in *C. elegans*.

### The C-terminal Region of GEI-8 is Capable of Binding GST-NHR-60

In order to confirm functional relatedness of GEI-8 with NCoR/SMRT, we performed a binding assay of the GEI-8 C-terminal domains to GST-NHR-60. NHR-60 is a member of a diversified subfamily of nematode receptors related to HNF-4 alpha and is important for embryonic and early larval development [24]. Mammalian HNF-4 alpha interacts both physically and functionally with SMRT [25] raising the possibility that NHR-60 may similarly interact with GEI-8. We divided the C-terminal region of *gei-8* into three domains: I. containing the NR1 binding site (position 2480–3485 in *gei-8a* isoform), II. containing the sequence between NR bindings sites (position 3413–4389) and III. containing the NR2 binding site (position 4274–5513). As expected from our sequence homology analysis of GEI-8 as it relates to NCoR/SMRT, the C-terminal region I of GEI-8 that includes the predicted NR1 binding site showed affinity to GST-NHR-60 but not to the control protein expressing the GST anchor used for pull-down experiments **(Figure S1)**.

### *gei-8* Expression

The *C. elegans gei-8* gene is located on chromosome III and gives rise to three predicted isoforms with mRNAs ranging from 5.3 to 5.6 kb (WormBase WS195). All predicted isoforms contain two SANT domains that could provide DNA and HDAC interaction functions **(** **Figure 2A and B** **).** Using primers based on predicted cDNA sequences of *gei-8* isoforms, we cloned three overlapping regions corresponding to *gei-8* cDNAs and confirmed the expression of predicted isoform *gei-8a* containing both SANT domains and two putative CoRNR-box like motifs **(** **Figure 2A** **).** The *gei-8a* cDNA clones also revealed that exon 12 can be removed and exon 16 is modified by alternative splicing **(** **Figure 2A** **)**; a spliced region of the same location and size as our cDNA clone was also detected by polyA mRNA expression profiling [26]. Depending on the presence or absence of exon 12, the size of *gei-8* cDNA is 5043 bp (*gei-8d*) and 5292 bp (*gei-8e*), giving rise to either a 1680 or 1763 amino acid long GEI-8 isoforms. We have not cloned the region containing the complete predicted protein encoded by inclusion of exon 16, however, polyA mRNA expression profiling data suggest that this variant is expressed. We confirmed the transcription of the *gei-8a* 5′ untranslated region (5′ UTR) and its trans-splicing to SL1 by PCR assays [27]. Expression of *gei-8b* and *gei-8c* was not detected using primers directed at predicted exons 1 to 3; our results are consistent with polyA mRNA expression profiling data generated by modENCODE **(** **Figure 2B** **)** [**26**].

**Figure 3:**
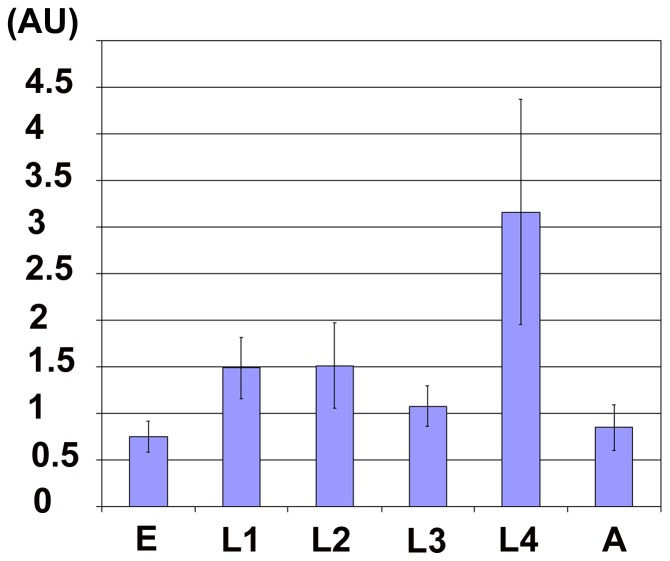
Normalized expression of *gei-8a.* The expression of *gei-8a* was measured for two regions (with primers 6168 and 01/042 and 6200 and 01/153) and quantitated relative to the constitutive gene *ama-1.* The expression of *gei-8a* peaks in the L4 stage. Relative expression was determined as proportion of lowest expression found in the embryonic stage and indicated as arbitrary units.

We quantified *gei-8* expression in individual embryonic and larval stages by real-time qPCR using cDNA prepared from synchronized populations of wild-type animals. We separately analyzed a region common for all predicted isoforms (*gei-8a, b, c*) as well as a *gei-8a-*specific region. We detected expression after probing both regions in all developmental stages at constant relative levels with the exception of the fourth larval (L4) stage where we observed a 2-fold increase for both **(** **Figure 3** **)**. We concluded that *gei-8a* was expressed throughout development, with its late larval increase possibly reflecting expression in the maturing germline.

The spatial expression pattern of *gei-8* was studied using three different *gei-8::gfp* constructs based on the predicted start of transcription for *gei-8b* (promoter 1), the detected start of transcription for *gei-8a* (promoter 2), and an overlapping region covering both promoters (promoter 3) **(** **Figure 2C** **)**. pPD95.69 and pPD95.67 promoterless, nuclear localization signal-containing vectors were used for the promoter 1 and promoter 2 constructs, respectively. Expression from promoter 3 was studied by the PCR fusion-based SOEing approach [28].

**Figure 4:**
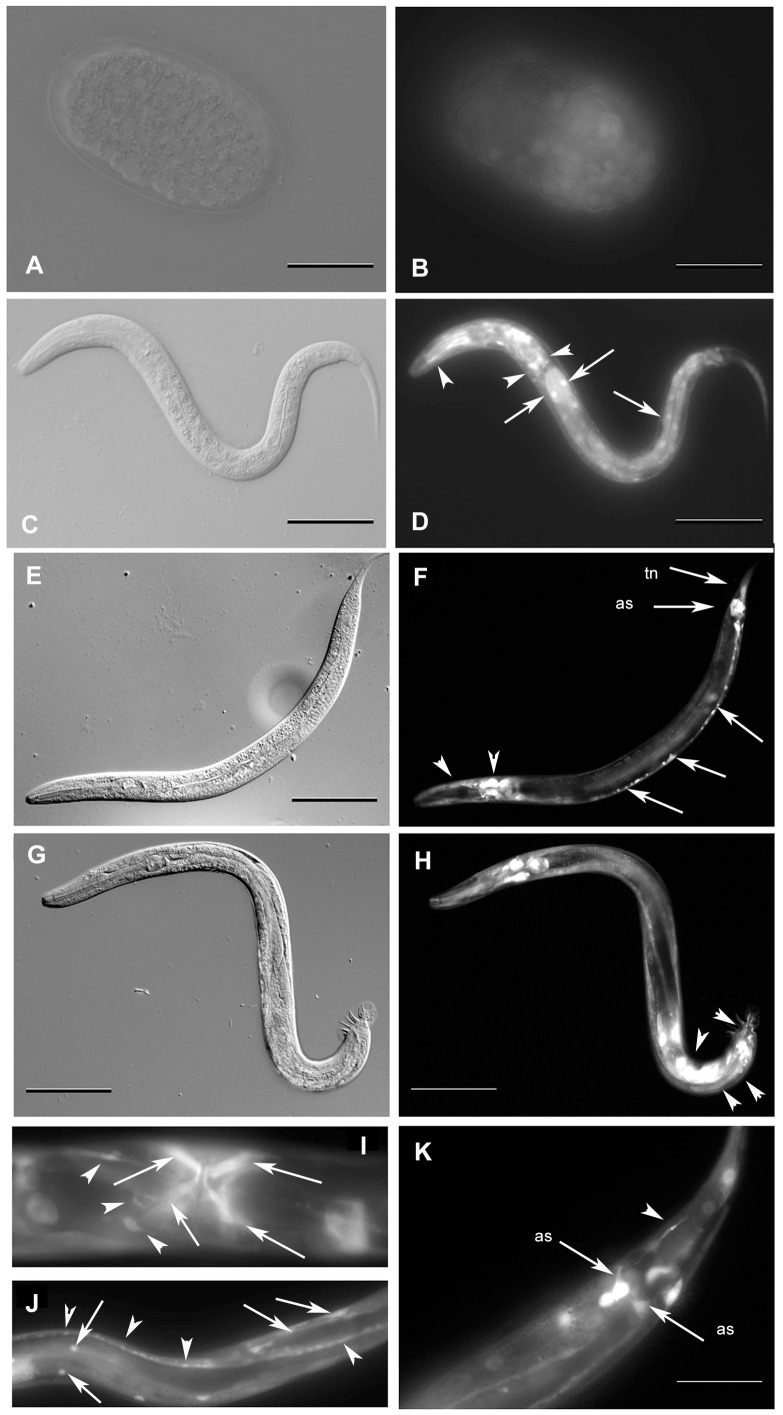
Analysis of *gei-8* expression using transgenic lines. The expression of *gei-8* was studied using transgenic lines carrying three different predicted promoters (#1, #2 and #3) fused with gene coding for GFP (indicated in Figure 2C) *gei-8::GFP*. Panels B and D show the expression from promoter #1 and panels F, H, I, J and K show the expression from promoter #3. Expression from promoter #2 construct was identical with that from promoter #3 and is not shown. (**A** and **B**) Embryonic GFP expression is ubiquitously present since comma stage. (**C** and **D**) L2 larva expressing *gei-8::GFP* ubiquitously with the highest expression in the head neurons and in the neuronal ring (arrowheads) and intestinal cells (arrows). (**E** and **F**) Expression of GEI-8::GFP in pharyngeal neurons (arrowheads), ventral nerve cord (arrows), anal sphincter (arrow - as) and tail neurons (arrow - tn) of an L4 larva. (**G** and **H**) Expression of GEI-8::GFP in L4 male larva. Additional expression is seen in male specific neurons (arrowheads). (**I**) L4 larva expressing GEI-8::GFP in egg laying structures, vulval and uterine muscles (arrows), egg laying neurons (arrowheads). (**J**) GEI-8::GFP expression in somatic muscles (arrows) and nerve cord (arrowheads). (**K**) Detail of expression of GEI-8::GFP in hermaphrodite tail neuron (arrowhead) and anal sphincter (arrows - as). (Figs. **A**, **C**, **E**, **G** in Nomarski optics and **B**, **D**, **F**, **H**, **I**, **J**, **K** in fluorescence microscopy). Scale: **A**, **B**, **I**, **J** 20 µm; **C**, **D**, **E**, **F**, **G**, **H** 100 µm; **K** 50 µm.

The promoter 1 reporter gene consisted of 1.8 kb upstream of the predicted *gei-8b* start codon and 222 bps of predicted exon 1. Its expression started in embryos at the comma stage in a ubiquitous pattern and was present in all larval stages. In larvae, the expression was detected in pharyngeal and tail neurons, intestinal cells, egg-laying muscles and the anal depressor **(** **Figure 4** **)**. The promoter 2 reporter gene construct consisted of 2.3 kb upstream of the predicted *gei-8a* start codon and included exon 1 and 64 bp of exon 2. The expression of this reporter gene was observed in all larval stages starting at the L1 stage and continuing through adulthood where expression was primarily observed in neurons of the pharyngeal nerve ring, head neurons, tail neurons and the egg-laying muscles**.** The promoter 3 reporter gene construct contained 6.2 kb upstream of the predicted *gei-8a* start codon, covering both promoter regions 1, 2 and exons 1, 2 and a part of exon 3; GFP sequences were derived from pPD95.75 by SOEing [28] and did not contain a nuclear localization signal. Expression of this reporter gene started at the embryonic comma stage. Larval expression was detected in pharyngeal neurons, ventral and dorsal nerve cords, tail neurons, egg-laying neurons, and egg-laying muscles. In males, GFP was observed in male-specific tail ganglia and rays. Typical examples of GEI-8::GFP cell- and tissue-specific expression are shown in **Figure 4** **.** Taken altogether, our reporter gene expression results defined multiple and distinct cis-acting regulatory regions of *gei-8* that drive similar expression patterns that are present throughout development and predominantly in neurons. Expression in the germline would not be revealed by this strategy because transgenes are usually silenced in the germline [29]. However, we noted that *gei-8* expression in the germline has been detected by Y. Kohara’s in situ hybridization results accessible in the Kohara in situ database NEXTDB (http://nematode.lab.nig.ac.jp).

### Loss of *gei-8* Results in Mutant Phenotypes

We obtained the VC1213 strain harboring a *gei-8(ok1671)* deletion allele generated by the *C. elegans* Knockout Consortium. The mutation was initially characterized as a 1095 bp deletion/45 bp insertion affecting exons 7 and 8 of *gei-8a,* removing the intron between them. We verified the size and location of the deletion by PCR genomic amplification from mutant animals and showed that the inserted sequences are identical to a 45 bp region from exon 7 starting at position 1550 of the predicted *gei-8a* isoform cDNA sequence. Sequencing the *gei-8(ok1671)* cDNA revealed a stop codon present in the *gei-8(ok1671)* transcript at position 663, giving rise to a predicted protein containing SANT1 and SANT2 domains, but missing the majority of the putative NR interaction sites at the C-terminus of the protein. The mutant mRNA was detected in homozygous *gei-8(ok1671)* animals using RT-PCR at levels similar to wild-type animals, suggesting the premature stop codon may be bypassed in some transcripts by alternative splicing or that the premature stop codon is not efficiently recognized by nonsense mediated decay [30]. Thus, truncated GEI-8 protein may be present in homozygous mutant larvae.

**Figure 5:**
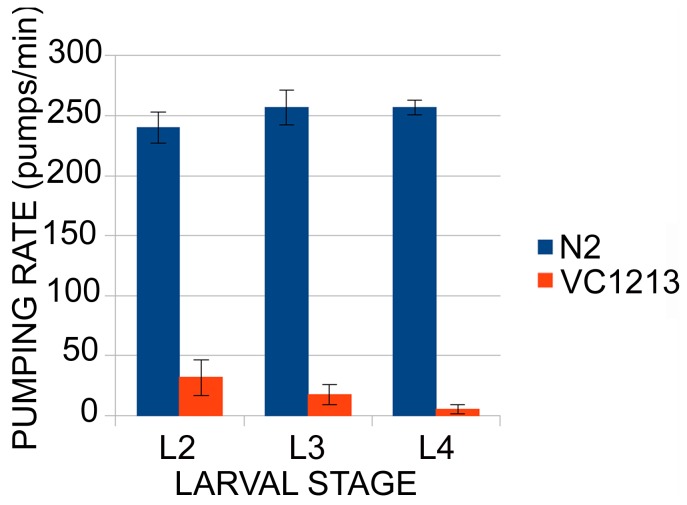
Analysis of the pharyngeal pumping rate of *gei-8(ok1671)* mutant animals and controls. Pharyngeal pumping rate is regulated by cholinergic transmission. In *gei-8* mutants the pumping rate is low compared to wild-type animals and decreases with age (n = 10 for each category).

**Figure 6:**
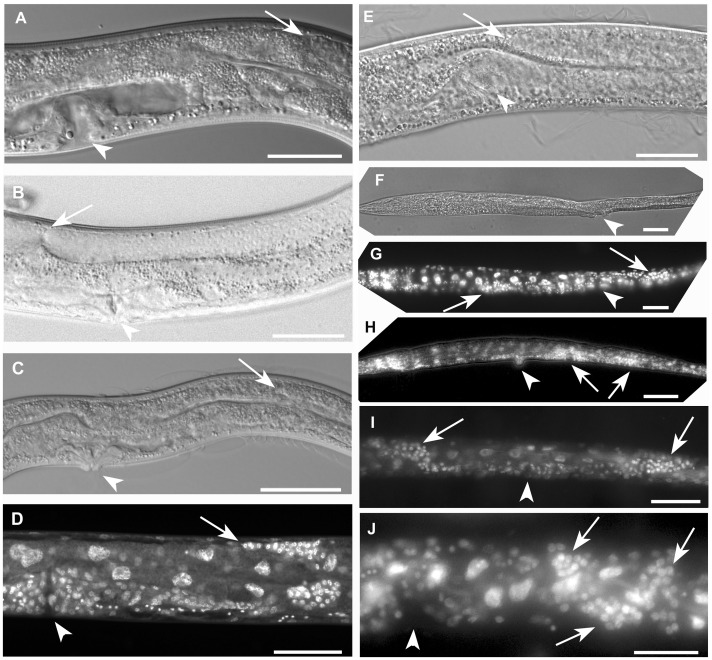
Development of the germline in *gei-8(ok1671)* mutants and additional phenotypic changes induced by RNAi targeted against Y9C9A.16 (*sqrd-2*) in homozygous *gei-8(ok1671)* mutants. (**A**) The reproductive structures of a wild-type larva at the L4 stage is shown. The vulva is indicated by an arrowhead and formation of the uterus is visible next to vulval structures. The position of the lead migrating cell for the gonad (distal tip cell) during the larval L4 stage is indicated by arrow. (**B**) Development of the gonad in a young adult N2 animal. The distal gonad arm continues in growth beyond the position of the vulva (marked by arrowhead) and makes contact with the proximal gonad arm (arrow). (**C**) *gei-8(ok1671)* mutant gonadogenesis by Nomarski optics. The arrested gonad arm in a position similar to wild type L4 larva is indicated by arrow. The vulva is marked by an arrowhead. (**D**) A *gei-8(ok1671)* mutant with arrested growth of the gonad as visualized by DAPI staining. The distal tip of arrested gonad is marked by an arrow and the vulva by an arrowhead. (**E**, **F**, **G**, **H**, **I** and **J**) Additional phenotypic changes induced by RNAi targeted against Y9C9A.16 (*sqrd-2*) region including three 21U-RNAs: 21ur-2020, 21ur-11733 and 21ur-9201 in *gei-8(ok1671)* homozygous mutant animals. (**E**) A *gei-8(ok1671)* mutant treated with *sqrd-2* RNAi shows growth of the gonad beyond the usual arrest point, reaching the position of the vulva (marked by arrow and arrowhead, respectively). (**F**) Additional phenotypes of *gei-8(ok1671)* animals treated with *sqrd-2* RNAi. Nomarski optics view of homozygous *gei-8(ok1671)* larva treated with *sqrd-2* RNAi revealing frequent growth defects, including irregular body shapes, (distention of proximal part of the body and thin elongation of the distal part of the body) and extended growth of the distal part of the gonad. The gonad is visualized by DAPI staining in panel **G** (distal arm of the gonad is marked by right arrow, proximal arm of the gonad is marked by left arrow). Arrowhead indicates the position of vulva in panels E, F and G. (**H**) Additional growth defects induced by *sqrd-2* RNAi in homozygous *gei-8(ok1671)* worms including a Pvul phenotype (arrowhead), accumulation of gonadal cells with a possible incomplete second vulva formation (left arrow) and a distal arm of germline that fails to turn and instead continues to grow in the direction of the thin and elongated tail (right arrow). (**I**) A mutant animal with germline growth directional changes of both gonad arms induced by *sqrd-2* RNAi: anterior gonad arm makes an incomplete turn dorsally and continues to grow in the anterior direction (left arrow) while the posterior gonad arm fails to turn and continues in additional growth towards the tail (right arrow). The position of vulva is indicated by arrowhead. (**J**) A homozygous *gei-8(ok1671)* mutant developing a convoluted irregular accumulation of cells of distal gonad arm in the position of gonad turn (marked by arrows). The position of vulva is indicated by arrowhead. Scale **A**, **B**, **D**, **E** and **J** 50 µm, **C**, **F**, **G**, **H** an **I** 100 µm.

The homozygous *gei-8(ok1671)* animals had obvious phenotypes, including a progressive defect in locomotion starting at the L2 stage that was marked by a delayed response to prodding and a low pharyngeal pumping rate **(** **Figure 5** **)**. Compared to wild-type animals of the same age, mutants were also characterized by a shorter maximum body length (750.25 µm, n = 6, SD = 50.59 µm), a convoluted intestine, gonadogenesis defects including loss of the spermathecae, sterility, and arrest at the L4 stage of development **(** **Figure 6** **C and D)**. After outcrossing the original mutant strain to wild-type animals, the heterozygous mutant strain segregated 26.2% (SD = 2.4; n = 2656) affected progeny as described **(** **Table 1** **)**. To verify that the observed phenotypes were caused by the *ok1671* deletion allele of *gei-8*, we performed rescue using intact *gei-8* genomic DNA. This method has been used previously to generate transgenic animals and to rescue mutant animals [31]–[34]. Overlapping PCR regions containing a 6 kb putative promoter region plus the complete coding region of *gei-8a* **(** **Figure 2D** **)** were injected into heterozygous *gei-8(ok1671)* animals along with pRF4 injection marker, rollers were selected and their progeny were screened for locomotion defects as defined as impaired responses to prodding. The wild-type *gei-8* genomic sequences were able to reduce the percentage of affected mutant progeny segregating from heterozygous hermaphrodites from 26.2% to 18.3% (SD = 3.4; n = 7883); this difference was significant using the Student's *t*-test (p<0.001; SD = 3.16) **(** **Table 1** **)**. Importantly, all other mutant phenotypes also showed improvement in the presence of wild-type genomic sequences leading us to conclude that most, if not all, of the defects we observed in *gei-8(ok1671)* animals were due to disruption of GEI-8 activity.

**Table 1:** Rescue experiment of *gei-8(ok1671)* with overlapping amplified regions of genomic DNA injected into the gonads of parents.

Target gene	Number of scored progeny	Affected larvae	%
Non-injected *ok1671*	2656	696	26.2 SD = 2.4
*gei-8* rescue after injections	7883	1443	18.3 SD = 3.16

We scored 20 *gei-8(ok1671)* mutant animals for germline development defects using Nomarski optics and DAPI (4',6-diamidino-2-phenylindole) staining of fixed animals. In 19/20 mutant animals examined, distal tip cell (DTC) migration stopped short, reaching only two thirds of it’s normal length of migration on the dorsal side of the animal **(** **Figure 6C and D** **)**. In homozygous mutant animals, both gonad arms were underdeveloped, containing fewer meiotic nuclei and germ cells compared to wild-type and heterozygous *gei-8(ok1671)* control animals. We also failed to detect spermathecae, sperm, or embryos in any mutant animals. We concluded that *gei-8(ok1671)* mutant germlines are arrested at the L4 stage, before complete gonad elongation and spermatheca development, although some somatic markers of early young adult stages were already present (adult alae, adult vulva)**.** The arrested animals also had a shorter lifespan than wild-type controls. The average lifespan of *gei-8(ok1671)* mutants at 20°C was 11 days (n = 21, SD = 3.4), which was significantly shorter than the average lifespan of wild type controls (17.4 days, n = 12, SD = 3.9).

Two muscle-related phenotypes were observed in homozygous *gei-8*(*ok1671*) mutants; decreased locomotion on plates and decreased pharyngeal pumping rates. The locomotion defects we observed for *gei-8(ok1671)* animals on plates prompted us to carry out a thrashing assay. When placed in liquid, wild-type animals bend back and forth moving their head and tail relative to the midbody of the animal in a thrashing motion that can be easily quantitated [35]. In the *gei-8(ok1671)* mutant strain, this natural thrashing behavior is impaired and deteriorated over the course of development. Unlike wild-type controls, homozygous *gei-8(ok1671)* mutants at the L4 stage were not able to perform smooth thrashing. Instead, their movements were spastic and irregular, averaging only 0 to 6 bends per minute at the L4 stage compared to about 250 bends per minute for wild-type animals (n = 10). Similarly, assays of pharyngeal pumping revealed irregular and reduced contraction rates in the homozygous mutants that became progressively worse with age. The average pumping rate in *gei-8(ok1671)* homozygous animals was 31.8, 17.5 and 5.3 pumps per minute at L2, L3 and L4 stages, respectively (n = 10 for each larval stage), compared to 250 pumps per minute for wild-type animals.

The movement and pharyngeal mutant phenotypes could be due to defects in the functioning of muscle, nerves, or both. To investigate muscle structure, we performed immunostaining using phalloidin and anti-MYO3 antibody directed against contractile apparatus components. Phalloidin stains actin filaments whereas the anti-MYO3 probe recognizes myosin heavy chain-3 [36], [37]. Immunostaining revealed no obvious structural differences between *gei-8(ok1671)* mutants and wild-type controls (not shown). Yamamoto et al. reported increased mitochondrial oxidative function in *C. elegans* after *gei-8* inhibition by RNAi [13]. We confirmed that finding using MitoTracker Red to visualize the mitochondrial oxidative state; homozygous *gei-8(ok1671)* mutants had an average mean density of staining that was more than 2.7 times greater (p<0.001) than that observed in wild-type larvae (**Figure S2**). Elevated MitoTracker staining could also be visualized in heterozygous *gei-8(ok1671)* mutants compared to wild-type N2 worms, but was not statistically significant in densitometric analysis of randomly selected progeny of heterozygous *gei-8(ok1671)* animals with a normal phenotype (which included both heterozygous mutants as well as wild-type animals (**Figure S3**).

**Figure 7:**
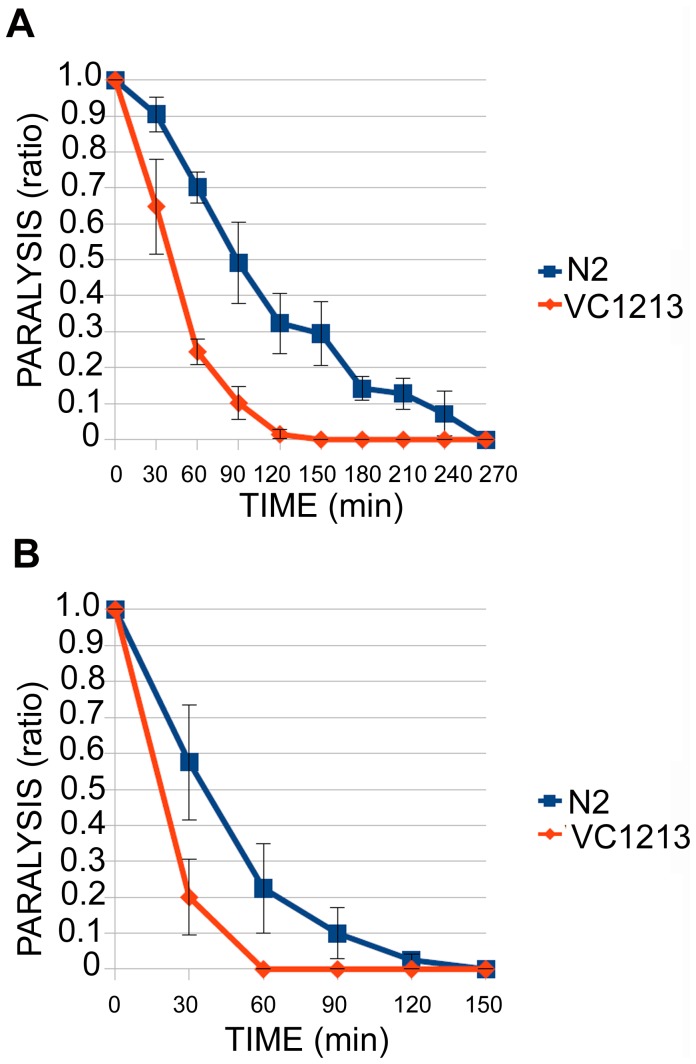
Analysis of neuromuscular function of *gei-8(ok1671)* mutant (VC1213). Aldicarb and levamisole sensitivity assays revealed increased sensitivity of *gei-8* mutants towards the acetylcholinesterase inhibitor aldicarb (**A**) and levamisole (**B**) suggesting a synaptic defect in cholinergic transmission.

The absence of obvious muscle defects in *gei-8* mutants suggested that the locomotion and pharyngeal pumping defects might be due to problems in neurotransmission. We investigated synaptic transmission by assaying animal sensitivity to either aldicarb or levamisole [38], [39]. Aldicarb is a reversible acetylcholinesterase inhibitor that increases the accumulation of acetylcholine in the synaptic cleft causing whole body paralysis and inhibition of pharyngeal pumping. Homozygous *gei-8(ok1671)* mutants (n = 64) and wild-type animals (n = 75) at the L4 stage were incubated on NGM plates with 1 mM aldicarb and scored over time for paralysis in three separate experiments. The onset of paralysis occurred significantly earlier in *gei-8(ok1671)* mutants than in wild-type controls **(** **Figure 7A** **)**. Levamisole is a cholinergic agonist that also results in animal paralysis. We performed two experiments with homozygous *gei-8(ok1671)* mutants (n = 40) and wild-type animals (n = 40) at the L4 stage on NGM plates with levamisole at a concentration of 1 mM. As in the aldicarb assay, the onset of paralysis occurred significantly earlier in *gei-8(ok1671)* mutants compared to controls **(** **Figure 7B** **)**. Taken together, these results indicate that the *gei-8(ok1671)* mutation results in abnormal cholinergic signaling, however, it does not distinguish between post-synaptic versus pre-synaptic transmission defects.

### *gei-8* Loss of Function Leads to Transcription Deregulation

Effects of the *gei-8(ok1671*) mutation on gene expression were studied with whole genome microarrays (Affymetrix). Changes in gene expression were defined as increased or decreased if statistically significant compared to wild-type controls in at least 2 out of 3 biological replicates. Deregulated genes were analyzed for Gene Ontology (GO) term enrichment and clustered according to functional classification using DAVID 6.7 [40] and KEGG pathway tools [41].

Expression microarray analysis revealed 756 probe sets with decreased expression, corresponding with 690 unique Wormbase IDs **(Table S1)**. DAVID classification tools [40] identified 645 IDs using medium classification stringency. GO analysis resulted in 32 clusters with an enrichment score greater than 2 and P<0.05. The list was enriched in spliceosome (29 genes), proteasome (13 genes), cysteine and methionine metabolism (7 genes), and RNA polymerase genes (6 genes) as identified by KEGG pathway analysis. Among specific genes involved are RNA polymerase II and III (Pol II subunits B4, B7, B9 and Pol III subunits AC2 and F09F7.3), spliceosome components (U1 to U6 snRNAs, *hel-1* helicase and others), and proteasome subunits (*pas-3*, *pas-4*, *pbs-1*, *pbs-3*, *pbs-4*, *pbs-6*, *pbs-7*, *rpt-1, rpt-2*, *rpn-2*, *rpn-5*, *rpn-8*, *rpn-12*). The most common functional categories over represented by the changes in gene expression were growth, embryonic or larval development and development of reproductive structures. Other clusters include multiple histones and histone-like genes, mitochondrial membrane proteins, sperm structural proteins and hedgehog-like family genes. Interestingly, the set of genes downregulated in *gei-8* mutants included several genes required for proper muscle function, including *unc-52* (myofilament assembly and/or attachment of the myofilament lattice to the cell membrane), *unc-27* (troponin I family), *unc-54* (muscle myosin class II heavy chain), *pat-10* (body wall muscle troponin C), *lev-11* (tropomyosin), *mlc-2* (myosin light-chain), and *tni-1* (troponin 1). It is unclear if such changes in muscle gene expression contribute to, or are the result of, the defective movement phenotypes we observed in *gei-8(ok1671*) mutant animals. Depletion of NCoR1 function specifically in mouse muscle resulted in increased muscle mass and mitochondrial function [13], a phenotype opposite to what we observed in worms with reduced GEI-8 activity in all tissues.

Microarray analysis revealed 296 probe sets with increased expression, corresponding to 275 unique Wormbase IDs (**Table S2**). GO analysis identified 7 clusters with an enrichment score greater than 2 and P<0.05. Enriched clusters included gene annotations for life span and aging, lipid transport and vitellogenin genes, stress response (heat shock and cellular stress), metabolic genes (sugar metabolism, glycolysis), and neuropeptide signaling (including genes coding for neuropeptide like proteins *nlp-27* to *nlp-32*). The KEGG pathway analysis identified six groups including genes involved in glycolysis (8 genes), cystein methionine metabolism (4 genes), galactose metabolism (3 genes), pentose phosphate pathway (3 genes), fructose and mannose (3 genes) and tryptophan metabolism (3 genes).

One of the most significantly affected genes in the *gei-8(ok1671)* homozygous mutants was Y9C9A.16, encoding a predicted mitochondrial sulfide:quinone oxidoreductase, which had an averaged 7.6-fold increase in expression compared to wild-type controls; this increase was confirmed by RT-qPCR. The Y9C9A.16 region is assayed by Affimetrix probe set 184710_at and, interestingly, includes three 21U-RNAs; 21ur-2020, 21ur-11733 and 21ur-9201. To determine if disruption of expression of Y9C9A.16 affected development, we performed RNAi targeted to the spliced mRNA covered by the Affymetrix probe set (184710_at) or only the regions that include 21ur-2020, 21ur-11733 and 21ur-9201. Progeny of parental animals injected with dsRNA targeting the specific regions were scored using Nomarski optics and fluorescent microscopy (DAPI stained). We were not able to identify any specific phenotype of Y9C9A.16 knockdown in wild type animals. However, because the expression from Y9C9A.16 showed a dramatic response to loss of GEI-8 activity, we thought there might be a biological connection between them. We predicted that knockdown of the expression from Y9C9A.16 locus in *gei-8 (ok1671)* homozygous mutants might revert or modify some of the observed phenotypes; the latter was observed. RNAi-mediated knockdowns targeted to the region covered by the 184710_at probe set and the region containing 21ur-2020, 21ur-11733 and 21ur-9201 induced additional phenotypes in the *gei-8(ok1671)* homozygous mutant background. Additional phenotypes included severe distal tip cell migration defects, irregular gonadal nuclei tumor like accumulation of germline cells and vulval protrusions observed in 13.9% of homozygous *gei-8(ok1671)* animals treated with Y9C9A.16 RNAi (n = 481) (**Figure 6E, F, G, H, I and J** **and** **Table 2**). Interestingly, Y9C9A.16 has a paralogue in the *C. elegans* genome, the gene *sqrd-1* (**s**ulfide:**q**uinone oxido**r**e**d**uctase). This gene encodes a protein that is identical in size (361 aa) to Y9C9A.16 sharing 266 identical amino acids in its sequence although the genes share very little DNA homology. SQRD-1 expression is regulated by *hif-1* in response to H_2_S and HCN [42], is involved in innate immunity and is associated with numerous 21U-RNAs. RNAi targeted to unique regions of the *sqrd-1* coding region, including four 21U-RNAs, resulted in changes in gonad arm migrations and an accumulation of germline cells (4.5% affected, n = 198) that were similar, although less severe, as those observed after Y9C9A.16 RNAi. We concluded that the paralogues encoded by Y9C9A16 and *sqrd-1*, and perhaps their associated 21U-RNAs, have overlapping roles during development of the germline that can be exacerbated by loss of GEI-8 activity.

**Table 2:** Induction of additional gonad and body shape phenotypes in homozygous *gei-8*(*ok1671*) mutant worms by RNAi directed against *sqrd-2* or *sqrd-1*.

Target gene	Screening method	Hermaphrodites injected	Homozygous larvae scored	Larvae with additional gonad and body shape defects
*sqrd-2*	Nomarski optics	24	211	40
*sqrd-2*	DAPI staining	15	270	27
Total for *sqrd-2*	Nomarski optics+DAPI staining	39	481	67 (13.9%)
*sqrd-1*	Nomarski optics	30	151	5
*sqrd-1*	DAPI staining	10	47	4
Total for *sqrd-1*	Nomarski optics+DAPI staining	40	198	9 (4.5%)

## Discussion

### GEI-8 is a NCoR/SMRT Orthologue with Developmental Roles in *C. elegans*

Our results demonstrate that GEI-8 is the *C. elegans* orthologue of the vertebrate NCoR/SMRT corepressors and that it has essential developmental and transcriptional functions throughout development. GEI-8 has the critical structural motifs necessary for corepressor functions, including the domains for HDAC interaction and activation. Moreover, it is able to interact physically with nuclear receptors through its C-terminal domain that is known to tether NCoR/SMRT to NRs [21], [43], [44]. The identification of the NCoR/SMRT homologue in *C. elegans* allows us to extend to invertebrates the conserved developmental functions of these important corepressors. Although such links had been previously suggested by the discovery of SMRTER in *Drosophila*, questions remained because SMRTER was significantly different from the majority of NCoR/SMRT paralogues that had previously been annotated [45]. While the HDAC interacting domain SANT1 is clearly present in *Drosophila* SMRTER (**Figure 1**), the second SANT domain is absent. In this respect, *C. elegans* GEI-8 is more closely related to vertebrate NCoR/SMRT-like NR corepressors than to SMRTER.

We further show that GEI-8 is required for normal development in *C. elegans* based on our studies of a *gei-8* deletion allele that severely truncates or inhibits the protein product. Although expressed, at least at the mRNA level, this mutant allele is predicted to lack the GEI-8 nuclear receptor interacting sites while expressing an mRNA that codes for the domains necessary for HDAC binding and activation. There is no evidence for dominant negative activity of this truncated product as heterozygous animals appear completely wild-type and the introduction of wild-type *gei-8* coding regions in transgenic animals partially rescue multiple mutant phenotypes. Therefore, the mutant phenotypes likely represent the loss of function effects for *gei-8*. Given the early and widespread onset of *gei-8* reporter gene expression in embryos, which is also detected by RT-qPCR, it is very likely that GEI-8 functions throughout development and in most, if not all, tissues. The lack of embryonic or early larval defects in homozygous mutants likely reflects the maternal load of *gei-8* gene products in the embryo. It is also possible that GEI-8 has multiple functions requiring different amount of GEI-8 activity, with demands for higher levels post-embryonically, including germline development.

The most significant phenotype observed in *gei-8* mutants is the late-L4 larval arrest, as revealed by the extent of gonadogenesis and DTC migration. One possibility is that this arrest reflects the depletion of maternally loaded *gei-8* products and that in the absence of GEI-8 activity, development and/or cellular processes fail to be executed properly. This interpretation would be consistent with the late developmental defects seen when other essential, maternally provided gene products are exhausted, such as the G1 cell cycle regulators [46]–[48]. The second most pronounced phenotype in the *gei-8(ok1671)* homozygous mutants is reduced pharyngeal pumping. It is unclear what defect(s) is responsible for this reduced pharyngeal rate given that it is a semi-autonomous action of the pharyngeal muscles that can be stimulated, but does not require neuronal input [49], [50]. One possibility was that food sensation mechanisms were compromised in the *gei-8(ok1671)* mutants; in the absence of food, the pumping rate of wild-type worms is similar to the rate we observed in the homozygous mutants. However, when tested we found that *gei-8(ok1671)* mutants exhibited spontaneous chemotaxis towards OP50 lawns until the mutants terminally arrested late in development demonstrating that food sensation mechanisms were intact. Another explanation of reduced pharyngeal pumping is diminished activity of the MC pharyngeal cholinergic neuron and/or its receptor, EAT-2 that regulate pharyngeal pumping in response to food [51], [52]. Such reduced cholinergic signaling is consistent with the sensitivity we observed for *gei-8(ok1671)* mutants to levamisole and aldicarb. Further experiments are needed to determine exactly which pathways are perturbed and the molecular basis for these aberrations.

### GEI-8 Regulates Transcription

Whole genome transcriptional analysis indicates that GEI-8 is required for proper gene expression. Its loss-of-function allele resulted in altered expression of a wide range of genes; genes with elevated expression as well as with decreased expression were identified. However, while GO annotation of many genes that showed decreased expression correlated with the observed phenotypes, genes with increased expression (that could be potentially de-repressed) failed to show an obvious correlation. It is interesting to note that among the set of genes that were decreased in mutant worms were several muscle specific genes. Thus, while bodywall muscle was normal by gross observations with normal appearance, there may be defects in this tissue in homozygous mutants.

The set of deregulated (increased) genes included neuropetide-like protein genes (*nlp-27* to *nlp-32*). The neuropetides that are generated from these genes fall in the subfamily characterized by the sequence YGGW [53] and are related to Aplysia APGW neuropeptides that regulate male reproductive functions [54]; the functional consequences of this, if any, are currently unknown. However, in agreement with results reported by Yamamoto et al. for mice [13], we have detected numerous metabolic genes in the set of genes with increased expression in homozygous mutant worms.

The set of increased genes included several clusters of metabolic genes involved in lipid transport, sugar metabolism, glycolysis, and amino acid metabolism. Several nuclear receptors may be involved in this metabolic regulation. The majority of *C. elegans* NRs show similarity to HNF4a but some may support metabolic functions dependent on PPARs in vertebrates, as shown for NHR-49 [55]. Moreover, GEI-8 loss of function may be similar in metabolic regulation as shown for SMRT with a single disabled NR site (ID1 or mRID1, respectively) [56], [57]. Interestingly, one of the genes that was increased in *gei-8(ok1671)* mutants (Y9C9A.16) encodes a sulfide:quinone oxidoreductase that we name *sqrd-2*. Our results demonstrate that *sqrd-2* and *gei-8* functions are genetically linked; inhibition of *sqrd-2* in homozygous mutants *gei-8(ok1671)* induces partial reversal of *gei-8* mutant phenotypes as well as additional phenotypic changes. We also demonstrated similar reduction-of-function phenotypes for the *sqrd-2* paralogue, *sqrd-1*. Both *sqrd* genes are associated with 21U-RNAs scattered throughout the non-coding regions. It is intriguing to speculate that the gene expression pattern of *gei-8* loss of function may be dependent on this class of regulatory RNAs. 21U-RNAs have been shown to be critical for sperm development and transposon silencing [58]. Both *sqrd* genes may be linked to their associated non-coding 21U-RNAs that may be localized in mitochondria as part of piRNA biosynthesis [59]. Changes in the mitochondrial compartment induced by *gei-8* inhibition as reported by Yamamoto et al. [13] and observed in our experiments on *gei-8(ok1671)* mutants may also involve piRNAs mediated regulation. The connection to the regulation by 21U-RNAs is supported by our findings that additional changes in the phenotypes of homozygous mutants *gei-8(ok1671)* are induced by RNAi targeted at *sqrd-1* gene. One of the three isoforms of *sqrd-1* is predicted to code for a protein with the same length as the protein derived from *sqrd-2* and both proteins show 74% identity in amino acid sequences suggesting that these proteins may substitute for each other in function. 21U-RNAs located in *sqrd-2* show approximately 50% identity in the conserved cores formed by 16 or 17 bases with piRNAs found in *sqrd-1.* The levels of these non-coding RNAs, like the activity of *sqrd-1* and *-2*, may be critical for gonad and/or germline development and metabolism. The early embryonic lethality of mice lacking NCoR1/SMRT as well as NCoR2 prevents us from assessing whether the role of GEI-8 in gonadogenesis is an evolutionarily conserved feature [3], [60]. Interestingly, it was recently found that individual 21U-RNAs are regulated by fork-head transcription factors [61]. Moreover, the fork-head factor FoxP1 regulates development in concert with SMRT [62]. These results raise the intriguing possibility that GEI-8 might be directly involved in the transcriptional regulation of 21U-RNAs.

## Materials and Methods

### Worm Strains

All strains were maintained as described [63] and were grown on standard NGM plates or, in case of RNA isolation, on NGM plates capped with 2% agarose. Wild-type animals were N2 (var. Bristol). The VC1213 strain, kindly provided by the *C. elegans* Gene Knockout Consortium, carried the *gei-8(ok1671)* allele over a *bli-4* and GFP-marked balancer chromosome; homozygous *gei-8(ok1671)* mutants are lethal. Prior to experiments, we outcrossed the VC1213 strain three times to wild-type males. *gei-8::gfp* transgenic lines were constructed by injecting *gei-8* promoter constructs into N2 hermaphrodites as described previously [64].

### Total RNA Isolation and cDNA Preparation

Wild-type *C. elegans* animals were grown on 2% agarose-capped NGM plates, washed with water and frozen at −80°C. After thawing the pellet was resuspended in 0.5 ml of resuspension buffer (0.5% SDS; 5% 2-mercaptoethanol;10 mM EDTA; 10 mM Tris/HCl (pH 7.5) with 15 µl of proteinase K (20 mg/ml)), vortexed for 60 s and incubated at 55°C for 60 min. The sample was phenol-chloroform extracted and ethanol-precipitated, dissolved in water and treated with 1 unit of DNAse (Promega, Madison, WI) per 1 µg of total RNA for 30 min at 37°C. After phenol-chloroform extraction and ethanol-precipitation RNA was resuspended in DEPC water. cDNA was synthesized from the isolated RNA using Roche Transcriptor High Fidelity cDNA Synthesis Kit (Roche, Basel, Switzerland) with poly-T and gene specific primer 6242 from the 3′ untranslated region (UTR) of *gei-8*, or using Superscript II kit (Invitrogen, Carlsbad, CA) with random hexamer primers, all according to protocols by manufacturers.

### RNA Interference

Y9C9A.16 dsRNA was synthesized using a 774 bp region of gDNA containing exons 2 to 6 amplified by primers 7501 and 7502 and cloned into pCRII vector (Invitrogen). Prior to in-vitro transcription by T7 or Sp6 polymerases, the construct was linearized. dsRNA was prepared by incubating ssRNAs at 70°C for 10 min and at 37°C for 30 min, followed by phenol-chloroform purification, ethanol-precipitation and dilution in DEPC water. dsRNA was injected into gonads of N2 wild-type hermaphrodites, heterozygous *gei-8(ok1671)* mutants, and homozygous wild-type progeny of heterozygous *gei-8(ok1671)* mutant parents. *sqrd-1* RNAi was prepared as mentioned above using primers 7605 and 7606.

### Immunostaining

L4 stage homozygous VC1213 mutants and N2 control animals were put on slides coated with poly-L-lysine, fixed in 5% paraformaldehyde, covered by a cover glass and incubated in a wet chamber for 30 minutes at room temperature. Freeze crack was performed after freezing the sample on dry ice for 5 minutes. Samples were placed in −20°C methanol followed by series of rehydration in methanol:TTBS (9∶1, 7∶3, 1∶1 and 1∶4 10 minutes each). Staining of actin filaments was done using phalloidin labeled with Alexa Fluor 488 (Molecular Probes, Eugene, OR). Samples were incubated with phalloidin (1∶500 dilution) for 40 min and then washed in TTBS three times. Samples were mounted with fluorescent mounting medium (DakoCytomation, Copenhagen, Denmark) and coated with nail polish.

Immunostaining of myosin filaments was performed using anti-MYO3 antibody [65]. After rehydration samples were incubated with anti-MYO3 mouse antibody (1∶200 dilution) for 24 hours at 4°C, then incubated with anti-mouse IgG antibody labeled with Alexa Fluor 568 (1∶400 dilution). Each incubation with antibody was followed by three TTBS washes. Samples were mounted as described above.

### Staining with 4',6-diamidino-2-phenylindole (DAPI)

L4 stage homozygous *gei-8(ok1671)* mutants and N2 control animals selected from progeny of injected mothers were put on slides coated with poly-L-lysine and 20 µl of water, covered by a cover glass and put on dry ice. Freeze crack was performed after freezing the sample on dry ice for 5 minutes. Samples were kept in −20°C methanol for 10 minutes, then stained with DAPI (20 µl, 1∶1000 dilution of 1 mg/ml) and mounted with fluorescent mounting medium (DakoCytomation, Copenhagen, Denmark) and coated with nail polish.

### Longevity Assays

Longevity assays were performed as described [66] with modification. Adult hermaphrodites were allowed to lay eggs for 4–5 hours. Homozygous *gei-8(ok1671* mutants (n = 21) and N2 controls (n = 12) were cultured at 20°C and transferred to a new plate every second day. The vitality of the animals was checked once per day. Death was defined as cessation of pharyngeal pumping or lack of response to prodding.

### Cloning

We used primers designed according to predicted sequences of *gei-8* isoforms *a*, *b* and *c* (WormBase WS195). Multiple regions were amplified by Accuprime polymerase (Invitrogen), cloned into TOPO pCRII, pCR4 or XL vectors (Invitrogen) and sequenced (Avant 3100). Selected clones are displayed in **Figure 2A and C**. Primer sequences are as follows: 107, 7149, 7144, and 6242. The sequences of all primers are in **Table S3**.

### Sequencing the *gei-8(ok1671)* Allele

The mutation in strain VC1213 was confirmed by single-worm PCR with primers 107 and 307 producing bands of expected sizes for mutant and wild-type worms. The nature of *gei-8(ok1671)* deletion was confirmed by sequencing these PCR fragments (Avant 3100).

### Aldicarb and Levamisole Sensitivity Assays

L4 *gei-8(ok1671)* homozygous mutants and L4 N2 wild-type animals were scored for aldicarb or levamisole sensitivity on NGM plates with 1 mM aldicarb [38] or 1 mM levamisole [39]. The assays were performed at room temperature and scored every 30 minutes until complete paralysis of all animals. Paralysis was defined as cessation of pharyngeal pumping and lack of response to prodding. The score was plotted as the ratio of moving animals to the total number of all animals on the plate.

### Locomotion Assays

Thrashing assays were performed in 15 µl of 1× PBS solution on non-adhesive slides. One thrash was defined as a complete swing of the head, for example from left to right and left again. L4 stage VC1213 mutant animals and N2 controls were compared. All worms were allowed to acclimate to the solution for one min prior to scoring. The total number of thrashes was counted in one minute intervals. The pharyngeal pumping rate was counted per minute in well-fed worms in the presence of food at 20°C and VC1213 and N2 controls were compared at the L2, L3 and L4 stages (n = 10 for each stage).

### Real-time PCR

Two regions of the *gei-8* gene were analyzed for expression using the LightCycler 480 and the LightCycler® 480 SYBR Green I Master kit (Roche Diagnostics, Basel, Switzerland). Region 1 was amplified by primers 6200 and 05/153. Region 2 was amplified by primers 6168 and 01/042. We performed two independent reactions for each region. Reaction conditions were as follows: 5 min pre-denaturation at 95°C followed by 45 cycles amplification (10 s at 95°C, 15 s at 59°C, 15 s at 72°C) and melting curve analyses (5 s at 95°C, 1 min at 65°C and then continuously increasing temperature up to 97°C (temp rate 0,2°C/s)). Data were processed by the LightCycler® 480 software version 1.5. Efficiency values reflected standard curve dilution series, which corresponded to gel-purified ethanol-precipitated PCR products. The Cp values of studied genes were normalized relative to the constitutive gene *ama-1* encoding the large subunit of RNA polymerase II [67], [68].

### Mutant Rescue

The *gei-8* promoter and coding sequence was amplified in two overlapping PCR products RES1 and RES2 with primers 6174 and 6173; 6158 and 6243, respectively. The size of the overlapping region was 191 bp. Both PCR products were mixed together to a final concentration 250 ng/µl and injected with the pRF4 *(rol-6(su1006))* dominant marker at 100 ng/µl in VC1213 mutant or wild-type adult hermaphrodites. Rollers were selected from the progeny of injected mothers and kept individually per plate at 16°C until they finished laying eggs. Total progeny were counted and scored for embryonic lethality and the number animals carrying the mutant phenotype [31]–[34].

### GFP Reporters

Transgenic lines expressing *gei-8::gfp* from putative promoter 1 contains 1832 bp upstream of the translational start codon and 222 bp of predicted exon 1 of *gei-8b* and *c*. Putative promoter 2 contains 2300 bp upstream of ATG. Promoter 1 was amplified using primers 01/021 and 4938. Promoter 2 was amplified using primers 5056 and 5060. Promoter fragments were cloned into the GFP vectors pPD95.69 and pPD95.67, respectively and injected into L4 hermaphrodites. Both constructs contained a nuclear localization signal.

Transgenic lines expressing *gei-8::gfp* from putative promoter 3 were created by a PCR fusion-based approach described by Hobert (2002). A 6.2 kb long putative promoter region of *gei-8* was amplified by primers 6228 and 6230. Primer 6230 contains an overhang complementary to the *gfp* sequence of the pPD95.75 vector. The second product, containing *gfp* and *unc-54* sequences was amplified by standard primers 6232 and 6233 using the pPD95.75 vector as template. The overlapping fusion PCR product was obtained by diluting the two products with water to 10–50 ng/µl and using a 1∶1 mixture as a template for a subsequent PCR reaction with nested primers 6229 and 6234. The PCR fusion product was diluted to a final concentration of 50 ng/µl, mixed with the injection marker *rol-6* at 50 ng/µl and injected in N2 adult hermaphrodite animals. GFP expression was selected for until stable expressing lines were established.

### Microarrays

*C. elegans* whole genome expression microarrays (Affymetrix, Santa Clara, CA) were used to profile gene expression in three independent replicates based on manually selected homozygous *gei-8(ok1671)* mutants and matched N2 wild-type larvae at the earliest stage when mutants can be easily recognized based on their movement phenotype. Microarray chip data was analyzed by Affymetrix MAS 5.0 suite software (1.6-fold change in mRNA expression) and Robust Multichip Average (RMA) (1.2-fold change in mRNA expression) as part of the Partek genomics suite software package, all with a p-value less than or equal to 0.05. The microarray data has been deposited in the NCBI’s GEO database (http://www.ncbi.nlm.nih.gov/geo) accession number GSE40127.

### Detection of Mitochondrial Activity by MitoTracker Labeling

Manually selected worms were transferred to a 10 µl drop of 10 µM MitoTracker Red CMXRos (Invitrogen, Molecular Probes) for 2 hr at room temperature (21°C) in PBS and were kept in dark. Worms were transferred in a drop of 20 µl PBS to NGM plate seeded with OP50 bacteria and kept in dark for 2 hr. Worms were then manually transferred to microscopic slides with agarose layer for fluorescent microscopy. For densitometric analysis, L4 larvae were analyzed using the Olympus BX60 microscope with a DP30 camera and pictures recorded at constant settings. For densitometric analysis, pictures of 12 larvae were used (four for each group, N2 wild-type larvae, mutant larvae *gei-8(ok1671)* determined by their moving phenotype and worms from the progeny of heterozygous *gei-8(ok1761)* that appeared normal). The total area of 149 000 µm^2^ was analyzed in total 40 selected areas (excluding areas for determination of background values) using the computer program ImageJ Version 1.42q (Rasband, W.S., ImageJ, U. S. National Institutes of Health, Bethesda, Maryland, USA, http://imagej.nih.gov/ij/, 1997–2011).

### GST Pull-down Assay

The complete coding cDNA of NHR-60 [24] (with the exception of the first methionine codon) was amplified by PCR using primers 10/44 and 10/45 and cloned in pGEX-2T vector (Amersham Pharmacia Biotech, Amersham, UK). The glutathione-S-transferase (GST) fusion protein was expressed in *Escherichia coli* (BL-21-strain). For control experiments, the protein domain of GST was expressed from the pGEX-2T empty vector. Overnight cultures of transformed bacteria obtained from a single bacterial colony were cultured in 400 ml of Luria-Broth culture medium containing 100 µg/ml ampicillin at 37°C overnight. Cultures with O. D. (600 nm) = 0.8 were induced using 1 mM isopropylthiogalactopyranoside (IPTG) and the cultures were cultivated at 20°C for an additional 5 hr prior to harvesting by centrifugation at 3300×g in a swing out rotor at 4°C for 10 min. The bacteria were washed twice in phosphate-buffered saline (PBS) and resuspended in 5 ml of PBS. Bacteria were lysed in 6 ml of Lysis buffer, (Bio-Rad Laboratories, Hercules, CA) supplemented with protease inhibitor (1× COMPLETE, Roche, Basel, Switzerland), incubated on ice for 10 minutes with intermittent vortexing and sonicated four times 10 sec. at 80% intensity (Sonicator UP100H, Hielscher Ultrasonics, Teltow, Germany). The lysates were centrifugated at 11180×g/4°C/10 min. The supernatant was removed and filtered using a 0.22 µm filter. Glutathione-agarose (Sigma-Aldrich, Saint Louis, MO) was prepared by swelling 0.01 g of beads in 1 ml PBS (137 mM NaCl, 2.7 mM KCl, 4.3 mM Na2HPO4, 1.45 mM KH2PO4, pH 7.5). The beads were collected by sedimentation and swelling completed by repeating the swelling step for an additional 5 min. The beads were then resuspended in 100 ul of PBS. The resulting slurry was used for binding of GST or GST-NHR-60. For purification of GST-NHR-60 and GST, 100 µl of slurry (containing 0.01 g of beads), and 300 µl of bacterial lysates were incubated for 30 minutes at 4°C with intermittent mixing (approximately every 4 minutes). The beads were washed four times in 1 ml of PBS Triton X-100 (1%) (Sigma-Aldrich, Saint Louis, MO). Beads were collected by sedimentation and resuspended in 500 µl of PBS. The resulting slurry was divided to four aliquots of 100 µl that were used for the binding assay.

The C-terminal domains of *gei-8a* coding for regions with the putative NR binding sites were amplified by PCR from cDNA and cloned in three constructs in pCR4 or pCRII TOPO-TA cloning vectors (Invitrogen, Carlsbad, CA). The three constructs marked as I, II, III were prepared using the following primers and positions in *gei-8a* isoform (Construct I: 7749, 7750; position 2480–3485, construct II: 7751, 7752; position 3413–4389; construct III: 7753, 7754; position 4274–5513). Constructs I and III included the predicted NR sites, NR1 and NR2 respectively.

^35^S-radiolabeled proteins were prepared using an in-vitro TNT T7/T3 coupled reticulocyte lysate system (Promega, Madison, WI) and 1.48 MBq of ^35^S-methionine (37 TBq/mmol) (Institute of Isotopes, Budapest, Hungary) in the final volume 50 µl. Ten microliters of the final TNT product was used for binding at 22°C for 30 minutes with intermittent mixing every 4 minutes. The beads were washed 3 times in 1 ml of PBS and resuspended in a final volume of 40 µl of PBS. Subsequently 5 µl of 2 × Laemmli Buffer and 1 µl of beta-mercaptoethanol were added, samples were boiled for 5 minutes and 25 µl were used for polyacrylamide gel electrophoresis and autoradiography. 10 µl of supernatant was used for determination of ^35^S-methionine in samples using the Liquid Scintillation Analyzer Tri-Carb 1600 TR (Packard, Meriden, CT) and Ultima-Gold scintillation cocktail (Perkin Elmer, Waltham, MA). For determination of input in binding experiments, 2 µl of in-vitro transcribed-translated product was resolved using polyacrylamide gel electrophoresis, transferred on Whitman 3M paper, dried and radioactivity determined in cut stripes containing the translated proteins but not the unincorporated ^35^S-methionine.

## Supporting Information

Figure S1 **Binding analysis of C-terminal domains of GEI-8 including the predicted NR binding sites to GST-NHR-60.** (A). Pull-down experiment of GST (lanes 1 to 3) and GST-NHR-60 (lanes 4 to 6) with in vitro translated proteins covering the C-terminal domains of GEI-8 (GEI-8*) cloned in three constructs: domain I (position 2480–3485 in *gei-8a*, lanes 1 and 4), domain II (position 3413–4389) lanes 2 and 5 and domain III (position 4274–5513) lanes 3 and 6. The domains I and III contain the predicted NR1 and NR2 binding sites, respectively. (B) Pulled-down radioactivity determined by scintillation detection in the fractions shown in panel A. GST-NHR-60 binds in vitro translated domain I of GEI-8 (lane 4 in panel A and the corresponding bar in panel B) supporting the functional similarity between GEI-8 C-terminal region and NCoR/SMRT. The figure presents one of two experiments that gave similar results.
(PDF)

Figure S2 **Elevated mitochondrial activity in mutant** ***gei-8(ok1671)*** **larvae.** Control wild-type N2 L4 larvae and progeny of heterozygous mutant in the same developmental stage were stained using MitoTracker staining as described (Materials and methods). Panels A, D, G, and J show N2 control animals; panels B, E, H, and K show progeny of a mutant heterozygous parent that lack the mutant phenotype and represent heterozygous or wild-type larvae; panels C, F, I and L show homozygous mutant larvae, from the same parent. Exposure time was 50 ms in panels A, B and C and 100 ms in all other panels. Panels A to F show the proximal part of larvae; panels G to I show the middle part of the larval bodies and panels J to L show the distal part of larvae. Elevated activity in homozygous animals is apparent in panels C, F, I and L. The scale bar represents 100 µm.
(PDF)

Figure S3 **Densitometric analysis of MitoTracker staining expressed in arbitrary units.** N2 wild-type animals, and the progeny of heterozygous mutant parents divided according to the mutant phenotype to phenotypically normal heterozygous (+/−) and phenotypically homozygous (−/−) animals were analyzed. Elevated staining by MitoTracker in homozygous mutant larvae is statistically significant in paired Student’s T-test compared to both N2 and morphologically unaffected progeny of heterozygous parents (p<0.01).
(PDF)

Table S1 **List of genes with decreased expression in** ***gei-8(ok1671)*** **homozygous mutants.**
(PDF)

Table S2 **List of genes with increased expression in** ***gei-8(ok1671)*** **homozygous mutants.**
(PDF)

Table S3 **Primers used in the study.**
(PDF)
