# Supplementary material for: GEI-8, a Homologue of Vertebrate Nuclear Receptor Corepressor NCoR/SMRT, Regulates Gonad Development and Neuronal Functions in Caenorhabditis elegans
Source: PLoS One. 2013 Mar 6;8(3):e58462. doi: 10.1371/journal.pone.0058462 (PMC3590189; doi:10.1371/journal.pone.0058462)
Supplement: Figure S1 — Binding analysis of C-terminal domains of GEI-8 including the predicted NR binding sites to GST-NHR-60. (A). Pull-down experiment of GST (lanes 1 to 3) and GST-NHR-60 (lanes 4 to 6) with in vitro translated proteins covering the C-terminal domains of GEI-8 (GEI-8*) cloned in three constructs: domain I (position 2480–3485 in gei-8a, lanes 1 and 4), domain II (position 3413–4389) lanes 2 and 5 and domain III (position 4274–5513) lanes 3 and 6. The domains I and III contain the predicted NR1 and NR2 binding sites, respectively. (B) Pulled-down radioactivity determined by scintillation detection in the fractions shown in panel A. GST-NHR-60 binds in vitro translated domain I of GEI-8 (lane 4 in panel A and the corresponding bar in panel B) supporting the functional similarity between GEI-8 C-terminal region and NCoR/SMRT. The figure presents one of two experiments that gave similar results. (PDF) [file pone.0058462.s001.pdf]

Figure S1

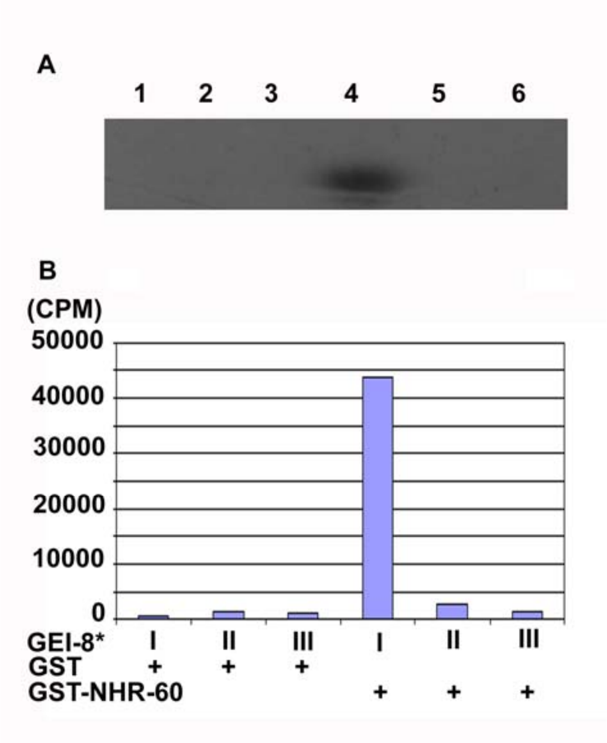

Figure S1. Binding analysis of C-terminal domains of GEI-8 including the predicted NR binding sites to GST-NHR-60. **(A).** Pull-down experiment of GST (lanes 1 to 3) and GST-NHR-60 (lanes 4 to 6) with in vitro translated proteins covering the C-terminal domains of GEI-8 (GEI-8*) cloned in three constructs: domain I (position 2480-3485 in *gei-8a*, lanes 1 and 4), domain II (position 3413-4389) lanes 2 and 5 and domain III (position 4274-5513) lanes 3 and 6. The domains I and III contain the predicted NR1 and NR2 binding sites, respectively. **(B)** Pulled-down radioactivity determined by scintillation detection in the fractions shown in panel A. GST-NHR-60 binds in vitro translated domain I of GEI-8 (lane 4 in panel A and the corresponding bar in panel B) supporting the functional similarity between GEI-8 C-terminal region and NCoR/SMRT. The figure presents one of two experiments that gave similar results.
