# Supplementary material for: GEI-8, a Homologue of Vertebrate Nuclear Receptor Corepressor NCoR/SMRT, Regulates Gonad Development and Neuronal Functions in Caenorhabditis elegans
Source: PLoS One. 2013 Mar 6;8(3):e58462. doi: 10.1371/journal.pone.0058462 (PMC3590189; doi:10.1371/journal.pone.0058462)
Supplement: Figure S2 — Elevated mitochondrial activity in mutant gei-8(ok1671) larvae. Control wild-type N2 L4 larvae and progeny of heterozygous mutant in the same developmental stage were stained using MitoTracker staining as described (Materials and methods). Panels A, D, G, and J show N2 control animals; panels B, E, H, and K show progeny of a mutant heterozygous parent that lack the mutant phenotype and represent heterozygous or wild-type larvae; panels C, F, I and L show homozygous mutant larvae, from the same parent. Exposure time was 50 ms in panels A, B and C and 100 ms in all other panels. Panels A to F show the proximal part of larvae; panels G to I show the middle part of the larval bodies and panels J to L show the distal part of larvae. Elevated activity in homozygous animals is apparent in panels C, F, I and L. The scale bar represents 100 µm. (PDF) [file pone.0058462.s002.pdf]

Figure S2

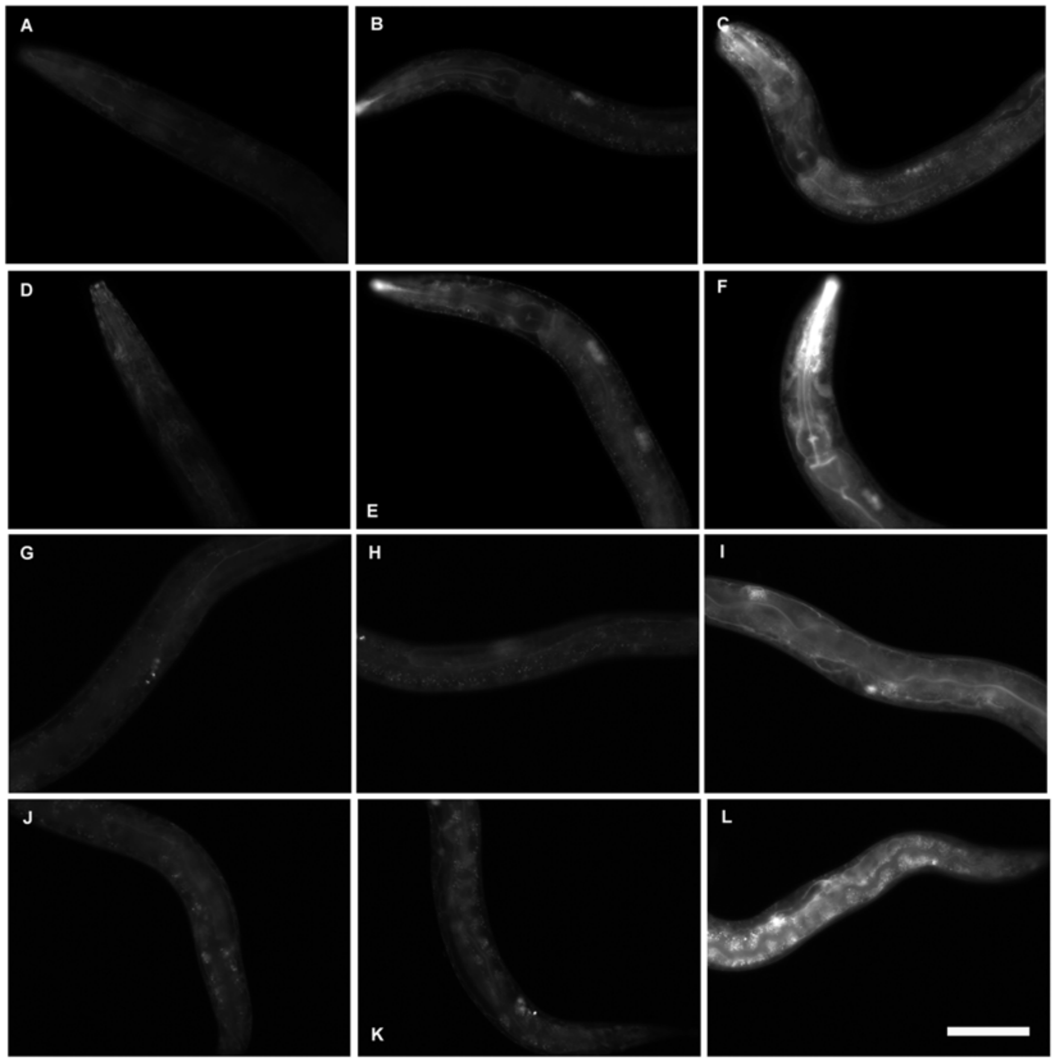

Figure S2. Elevated mitochondrial activity in mutant *gei-8(ok1671)* larvae. Control wild-type N2 L4 larvae and progeny of heterozygous mutant in the same developmental stage were stained using MitoTracker staining as described (Materials and methods). Panels A, D, G, and J show N2 control animals; panels B, E, H, and K show progeny of a mutant heterozygous parent that lack the mutant phenotype and represent heterozygous or wild-type larvae; panels C, F, I and L show homozygous mutant larvae, from the same parent. Exposure time was 50 ms in panels A, B and C and 100 ms in all other panels.

Panels A to F show the proximal part of larvae; panels G to I show the middle part of the larval bodies and panels J to L show the distal part of larvae. Elevated activity in homozygous animals is apparent in panels C, F, I and L. The scale bar represents 100 μm.
