# Supplementary material for: GEI-8, a Homologue of Vertebrate Nuclear Receptor Corepressor NCoR/SMRT, Regulates Gonad Development and Neuronal Functions in Caenorhabditis elegans
Source: PLoS One. 2013 Mar 6;8(3):e58462. doi: 10.1371/journal.pone.0058462 (PMC3590189; doi:10.1371/journal.pone.0058462)
Supplement: Figure S3 — Densitometric analysis of MitoTracker staining expressed in arbitrary units. N2 wild-type animals, and the progeny of heterozygous mutant parents divided according to the mutant phenotype to phenotypically normal heterozygous (+/−) and phenotypically homozygous (−/−) animals were analyzed. Elevated staining by MitoTracker in homozygous mutant larvae is statistically significant in paired Student’s T-test compared to both N2 and morphologically unaffected progeny of heterozygous parents (p<0.01). (PDF) [file pone.0058462.s003.pdf]

Figure S3

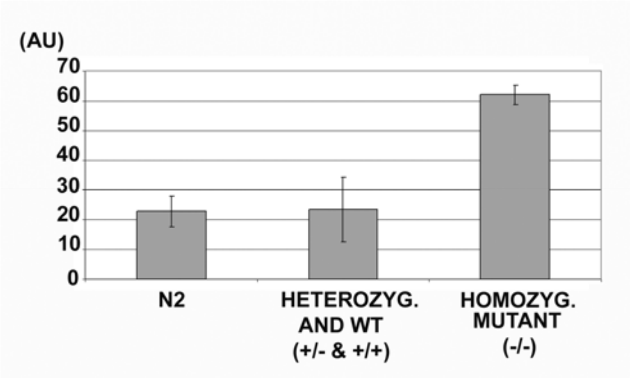

Figure S3. Densitometric analysis of MitoTracker staining expressed in arbitrary units. N2 wild-type animals, and the progeny of heterozygous mutant parents divided according to the mutant phenotype to phenotypically normal heterozygous (+/-) and phenotypically homozygous (-/-) animals were analyzed. Elevated staining by MitoTracker in homozygous mutant larvae is statistically significant in paired Student's T-test compared to both N2 and morphologically unaffected progeny of heterozygous parents ($p < 0.01$).
