# Supplementary material for: GEI-8, a Homologue of Vertebrate Nuclear Receptor Corepressor NCoR/SMRT, Regulates Gonad Development and Neuronal Functions in Caenorhabditis elegans
Source: PLoS One. 2013 Mar 6;8(3):e58462. doi: 10.1371/journal.pone.0058462 (PMC3590189; doi:10.1371/journal.pone.0058462)
Supplement: Table S1 — List of genes with decreased expression in gei-8(ok1671) homozygous mutants. (PDF) [file pone.0058462.s004.pdf]

Table S1. List of genes with decreased expression in *gei-8(ok1671)* homozygous mutants.

Gene WB ID	Gene Public Name	Oligo Set
WBGene00003825	ntl-2	171729_x_at
WBGene00019357	cpg-8	171743_x_at
WBGene00018967	F56D2.6	171760_x_at
WBGene00004702	rsp-5	171761_x_at
WBGene00017756	F23F12.12	171769_x_at
WBGene00006585	tni-3	171792_x_at
WBGene00020039	R12E2.14	171795_x_at
WBGene00018849	F55A3.3	171809_s_at
WBGene00021427	Y38F2AR.9	171817_x_at
WBGene00021004	W03F9.10	171823_x_at
WBGene00004699	rsp-2	171835_x_at
WBGene00003443	msp-50	171867_x_at
WBGene00004706	rsr-1	171871_x_at
WBGene00007192	B0491.5	171908_x_at
WBGene00009092	F23H12.2	171936_s_at
WBGene00006529	tba-2	171948_x_at
WBGene00004302	ran-1	171953_x_at
WBGene00000381	cct-6	171963_x_at
WBGene00001037	dnj-19	171968_x_at
WBGene00020588	T19H12.2	171979_x_at
WBGene00008053	cdc-48.2	172014_x_at
WBGene00002998	lin-9	172019_x_at
WBGene00001893	his-19	172055_x_at
WBGene00001894	his-20	172055_x_at
WBGene00000408	cdk-7	172068_x_at
WBGene00001023	dnj-5	172072_x_at
WBGene00022099	Y69A2AR.28	172076_x_at
WBGene00003370	mlc-2	172100_x_at
WBGene00022005	Y59H11AM.1	172113_x_at
WBGene00022164	Y71H2AL.1	172177_x_at
WBGene00012766	Y41E3.8	172203_x_at
WBGene00012781	nspd-7	172254_x_at
WBGene00008356	nspc-20	172268_x_at
WBGene00012489	Y19D2B.1	172280_x_at
WBGene00015593	C08E3.1	172309_x_at
WBGene00009638	nspc-14	172324_x_at
WBGene00009639	nspc-15	172324_x_at
WBGene00009637	nspc-13	172334_x_at
WBGene00023493	nspc-12	172334_x_at
WBGene00020033	R12E2.7	172346_x_at

WBGene00015339	C02E7.6	172353_x_at
WBGene00009634	nspc-9	172357_x_at
WBGene00009640	nspc-10	172357_x_at
WBGene00022754	nspd-1	172381_x_at
WBGene00017691	ilys-5	172400_x_at
WBGene00009259	F29G6.3	172455_x_at
WBGene00020715	nspd-4	172486_x_at
WBGene00020040	R12E2.15	172497_x_at
WBGene00009880	F49C12.11	172539_x_at
WBGene00017386	nspd-5	172546_x_at
WBGene00020039	R12E2.14	172547_x_at
WBGene00015605	C08E3.13	172565_x_at
WBGene00019168	H06I04.3	172617_x_at
WBGene00001924	his-50	172618_x_at
WBGene00013652	Y105C5B.12	172620_x_at
WBGene00013636	Y105C5A.12	172627_x_at
WBGene00022002	Y59E9AR.7	172629_x_at
WBGene00001895	his-21	172641_x_at
WBGene00001935	his-61	172649_x_at
WBGene00001892	his-18	172650_x_at
WBGene00001920	his-46	172679_x_at
WBGene00003462	msp-74	172687_x_at
WBGene00001910	C50F4.6	172692_x_at
WBGene00001936	his-62	172702_x_at
WBGene00009982	F53F1.4	172706_x_at
WBGene00009637	nspc-13	172713_x_at
WBGene00023493	nspc-12	172713_x_at
WBGene00003429	msp-31	172714_x_at
WBGene00009638	nspc-14	172715_x_at
WBGene00009639	nspc-15	172715_x_at
WBGene00009634	nspc-9	172716_x_at
WBGene00009640	nspc-10	172716_x_at
WBGene00003431	msp-33	172724_x_at
WBGene00001933	his-59	172732_x_at
WBGene00003587	ned-8	172739_at
WBGene00019026	F58A6.9	172743_x_at
WBGene00001922	his-48	172758_x_at
WBGene00001877	his-3	172763_x_at
WBGene00001881	his-7	172764_x_at
WBGene00003446	msp-53	172770_x_at
WBGene00003450	msp-57	172771_x_at
WBGene00001929	his-55	172774_x_at
WBGene00001938	his-64	172787_x_at
WBGene00001937	his-63	172788_x_at
WBGene00022760	ZK546.3	172798_x_at
WBGene00009983	F53F1.5	172799_x_at
WBGene00001931	his-57	172823_x_at
WBGene00003435	msp-40	172834_x_at
WBGene00013237	Y56A3A.19	172842_x_at

WBGene00003425	msp-10	172852_x_at
WBGene00003432	msp-36	172853_x_at
WBGene00003464	msp-77	172854_x_at
WBGene00003426	msp-19	172857_x_at
WBGene00001887	his-13	172871_x_at
WBGene00001906	his-32	172881_x_at
WBGene00003424	msp-3	172882_x_at
WBGene00003443	msp-50	172885_x_at
WBGene00003438	msp-45	172889_x_at
WBGene00001932	his-58	172891_x_at
WBGene00003467	msp-81	172893_x_at
WBGene00003442	msp-49	172896_x_at
WBGene00003470	msp-152	172900_x_at
WBGene00003448	msp-55	172903_x_at
WBGene00003465	msp-78	172905_x_at
WBGene00003466	msp-79	172906_x_at
WBGene00003449	msp-56	172907_x_at
WBGene00001996	hpl-2	172919_x_at
WBGene00003468	msp-113	172922_x_at
WBGene00003452	msp-59	172925_x_at
WBGene00003458	msp-65	172927_x_at
WBGene00003444	msp-51	172928_x_at
WBGene00004917	snr-4	172931_x_at
WBGene00003434	msp-38	172934_x_at
WBGene00003463	msp-76	172938_x_at
WBGene00011743	T13F2.2	172941_x_at
WBGene00009078	rpb-12	172963_x_at
WBGene00011235	R11A5.7	172989_s_at
WBGene00001746	gsk-3	172990_at
WBGene00013209	bub-3	172992_s_at
WBGene00001716	grl-7	173004_s_at
WBGene00004143	pqn-59	173018_s_at
WBGene00004502	rpt-2	173034_s_at
WBGene00017643	czw-1	173041_s_at
WBGene00001690	grd-1	173042_s_at
WBGene00017691	ilys-5	173051_s_at
WBGene00011282	R74.8	173058_s_at
WBGene00001423	fib-1	173100_s_at
WBGene00003791	npp-5	173120_s_at
WBGene00019272	H42K12.3	173124_s_at
WBGene00010685	tag-216	173128_s_at
WBGene00004259	pyr-1	173131_at
WBGene00000549	cls-2	173133_s_at
WBGene00003920	par-5	173137_s_at
WBGene00003949	pbs-3	173141_s_at
WBGene00003953	pbs-7	173146_s_at
WBGene00001808	gut-2	173168_s_at
WBGene00020366	T08G2.3	173218_s_at
WBGene00001234	eif-6	173220_s_at

WBGene00019644	cpt-4	173238_s_at
WBGene00019537	K08D12.3	173240_s_at
WBGene00016655	acbp-1	173247_s_at
WBGene00001830	hcp-2	173260_s_at
WBGene00004387	rnp-4	173284_s_at
WBGene00002957	let-858	173299_s_at
WBGene00017300	F09F7.3	173320_s_at
WBGene00012126	T28D6.6	173324_s_at
WBGene00011155	R09B3.2	173336_s_at
WBGene00022075	Y69A2AR.3	173338_s_at
WBGene00001816	haf-6	173341_at
WBGene00022072	cpg-9	173348_s_at
WBGene00016918	C54E4.2	173350_s_at
WBGene00001064	dpy-2	173355_s_at
WBGene00004754	sec-23	173356_s_at
WBGene00017085	E01A2.2	173364_s_at
WBGene00022119	Y71F9AL.17	173371_s_at
WBGene00000160	apb-1	173376_s_at
WBGene00000377	cct-1	173382_s_at
WBGene00022127	yop-1	173394_s_at
WBGene00001077	dpy-18	173395_s_at
WBGene00003159	mcm-7	173401_s_at
WBGene00013077	ttr-24	173412_s_at
WBGene00001073	dpy-11	173425_at
WBGene00001840	hel-1	173455_s_at
WBGene00013284	Y57A10C.6	173472_s_at
WBGene00007696	tram-1	173546_s_at
WBGene00015512	C06A8.1	173556_s_at
WBGene00015734	C13B9.3	173566_at
WBGene00001716	grl-7	173567_s_at
WBGene00001235	elb-1	173581_s_at
WBGene00001005	dlc-1	173606_s_at
WBGene00013379	Y62E10A.13	173613_s_at
WBGene00003497	mup-4	173641_at
WBGene00001005	dlc-1	173665_at
WBGene00003800	npp-14	173670_s_at
WBGene00017799	F25G6.8	173678_s_at
WBGene00003062	lpd-6	173689_s_at
WBGene00001040	dnj-22	173712_s_at
WBGene00003406	mrg-1	173721_at
WBGene00020700	T22F3.11	173729_at
WBGene00002077	imb-3	173771_s_at
WBGene00006481	tag-135	173852_s_at
WBGene00009138	F25H9.6	173860_s_at
WBGene00004501	rpt-1	173894_s_at
WBGene00020216	trap-2	173896_s_at
WBGene00010738	K10D3.4	173944_at
WBGene00000776	cpl-1	173990_s_at
WBGene00002061	ife-3	173994_at

WBGene00019510	K07H8.10	174001_s_at
WBGene00007145	B0334.5	174046_s_at
WBGene00016746	C48B6.10	174061_s_at
WBGene00015920	C17G10.9	174065_at
WBGene00004174	pqn-95	174071_at
WBGene00018961	F56D1.3	174087_s_at
WBGene00001840	hel-1	174125_at
WBGene00013676	ekl-4	174179_s_at
WBGene00001498	frs-2	174181_s_at
WBGene00017328	F10C5.2	174296_at
WBGene00020281	T06A4.1	174378_at
WBGene00003962	pdi-1	174408_s_at
WBGene00020812	T25G12.5	174581_s_at
WBGene00019148	H03E18.1	174628_s_at
WBGene00003794	npp-8	174671_at
WBGene00002977	lev-10	174766_at
WBGene00020846	T27A10.6	174781_at
WBGene00015095	B0261.7	174782_s_at
WBGene00005018	sqt-3	174910_at
WBGene00010627	K07C5.4	174915_s_at
WBGene00004221	ptr-6	174995_s_at
WBGene00001072	dpy-10	175025_at
WBGene00018706	F52F10.2	175060_at
WBGene00022297	Y76B12C.3	175068_s_at
WBGene00000378	cct-2	175118_s_at
WBGene00015702	C11D2.4	175154_s_at
WBGene00001451	flp-8	175166_at
WBGene00018679	F52C12.2	175208_s_at
WBGene00004020	pho-1	175238_s_at
WBGene00001814	haf-4	175347_s_at
WBGene00018926	F56A11.6	175356_s_at
WBGene00001598	glh-1	175430_s_at
WBGene00013237	Y56A3A.19	175476_s_at
WBGene00004192	prx-2	175557_at
WBGene00003952	pbs-6	175565_s_at
WBGene00022287	Y75B7AR.1	175576_s_at
WBGene00019407	K05F1.5	175584_s_at
WBGene00000868	cyb-3	175681_at
WBGene00001620	glt-1	175715_s_at
WBGene00004042	plk-1	175834_s_at
WBGene00018782	cct-3	175916_at
WBGene00016238	C30A5.3	175939_s_at
WBGene00019678	K12H4.3	176010_at
WBGene00004918	snr-5	176049_at
WBGene00004187	prp-8	176091_s_at
WBGene00017319	F09G8.3	176095_at
WBGene00021506	Y41D4A.4	176096_at
WBGene00015591	C08C3.4	176105_s_at
WBGene00022489	Y119D3B.12	176137_at

WBGene00019537	K08D12.3	176145_at
WBGene00003794	npp-8	176153_at
WBGene00021420	trap-3	176161_at
WBGene00021427	Y38F2AR.9	176161_at
WBGene00022184	Y71H2AM.19	176198_at
WBGene00018366	F42H10.2	176265_at
WBGene00022176	Y71H2AM.11	176266_s_at
WBGene00022122	trap-1	176321_s_at
WBGene00021420	trap-3	176344_at
WBGene00021420	trap-3	176353_s_at
WBGene00002978	lev-11	176396_s_at
WBGene00006702	ubc-3	176411_s_at
WBGene00002047	icp-1	176457_at
WBGene00021470	tpxl-1	176457_at
WBGene00022415	Y102A11A.5	176459_at
WBGene00022412	Y102A11A.2	176467_at
WBGene00022415	Y102A11A.5	176467_at
WBGene00021849	Y54F10AM.5	176470_at
WBGene00003156	mcm-4	176475_s_at
WBGene00001232	eif-3.I	176491_at
WBGene00021847	Y54F10AL.1	176497_s_at
WBGene00021595	Y46E12BL.2	176525_s_at
WBGene00022103	cdh-12	176543_at
WBGene00022394	Y97E10AL.3	176590_at
WBGene00003947	pbs-1	176617_s_at
WBGene00021420	trap-3	176659_s_at
WBGene00019767	rpa-2	176701_at
WBGene00013728	Y111B2A.2	176730_at
WBGene00018921	sago-2	176756_s_at
WBGene00021782	Y51H7C.4	176788_at
WBGene00021335	spp-23	176939_at
WBGene00022069	eel-1	176995_at
WBGene00000665	col-90	177024_at
WBGene00021236	Y19D10B.7	177131_s_at
WBGene00006920	vha-11	177139_s_at
WBGene00003704	nhr-114	177144_at
WBGene00022164	Y71H2AL.1	177190_at
WBGene00001853	hil-2	177209_at
WBGene00021844	Y54E10BR.5	177239_s_at
WBGene00003129	map-1	177266_s_at
WBGene00000408	cdk-7	177297_s_at
WBGene00004178	prg-1	177402_s_at
WBGene00011548	T06G6.6	177505_at
WBGene00020115	R155.1	177535_s_at
WBGene00008603	F09B9.4	177555_at
WBGene00009287	F31C3.5	177574_at
WBGene00001830	hcp-2	177622_at
WBGene00014003	ZK593.3	177629_at
WBGene00012261	lpr-3	177650_at

WBGene00012255	W04G3.1	177724_at
WBGene00003576	ndc-80	177760_at
WBGene00022645	ZK6.11	177783_at
WBGene00008446	E01G4.3	177815_s_at
WBGene00009514	F37H8.5	177838_at
WBGene00007223	C01F6.9	177911_at
WBGene00012257	lpr-4	177916_at
WBGene00007353	C06A1.2	177971_at
WBGene00010266	dct-18	177978_at
WBGene00011736	T12D8.9	178078_at
WBGene00013858	ZC168.6	178080_at
WBGene00010044	F54C9.9	178192_at
WBGene00011107	R07E3.6	178194_at
WBGene00004143	pqn-59	178212_s_at
WBGene00003093	lys-4	178236_s_at
WBGene00007560	C14A4.9	178508_at
WBGene00011768	T14D7.2	178515_s_at
WBGene00008215	C49F8.3	178517_at
WBGene00077526	C25A1.16	178741_at
WBGene00011319	T01C3.2	178765_at
WBGene00011522	T06D8.1	178918_at
WBGene00006452	heh-1	178926_s_at
WBGene00008963	F19H8.2	178930_at
WBGene00007384	C06G8.1	179164_at
WBGene00008260	C52G5.2	179176_at
WBGene00013884	ZC412.3	179334_s_at
WBGene00005003	spp-18	179424_at
WBGene00001390	far-6	179591_at
WBGene00007996	C38C6.3	179600_at
WBGene00007999	tag-297	179686_at
WBGene00020472	lips-11	179688_at
WBGene00012544	Y37D8A.2	179851_at
WBGene00007016	mdt-15	179869_at
WBGene00019380	K04C2.2	179889_at
WBGene00018572	lin-42	179896_at
WBGene00019629	cid-1	179905_at
WBGene00015956	C18B2.5	180040_s_at
WBGene00004461	rpn-5	180117_s_at
WBGene00008915	F17C11.4	180312_at
WBGene00000928	dao-2	180375_at
WBGene00077490	M03A1.8	180375_at
WBGene00000928	dao-2	180376_s_at
WBGene00021026	W04C9.4	180426_at
WBGene00016133	C26B9.3	180439_at
WBGene00004219	ptr-4	180485_s_at
WBGene00004120	pqn-32	180505_at
WBGene00022680	ZK180.6	180664_s_at
WBGene00016918	C54E4.2	180685_at
WBGene00010409	H21P03.2	180788_at

WBGene00020550	T17H7.1	180889_at
WBGene00001037	dnj-19	180919_s_at
WBGene00016670	ilys-3	180946_at
WBGene00020209	T04C9.1	180969_s_at
WBGene00010351	H02I12.1	180976_s_at
WBGene00001694	grd-5	180979_at
WBGene00018337	F42A9.8	181002_at
WBGene00007601	C15C6.2	181008_at
WBGene00009573	F40E10.5	181113_at
WBGene00009926	noah-2	181136_s_at
WBGene00004989	spp-4	181156_at
WBGene00006713	ubc-18	181170_s_at
WBGene00017749	F23F1.9	181235_at
WBGene00017483	lgc-22	181326_at
WBGene00012543	nkcc-1	181421_at
WBGene00019332	K02F3.9	181450_at
WBGene00008035	C39E9.12	181453_at
WBGene00015803	C15H9.9	181509_at
WBGene00018950	F56C9.6	181578_at
WBGene00004998	spp-13	181681_at
WBGene00011831	T19B10.2	181714_at
WBGene00011831	T19B10.2	181715_s_at
WBGene00020149	T01D1.4	181722_s_at
WBGene00015646	mlt-10	181793_at
WBGene00008925	F17H10.1	181797_at
WBGene00022569	ZC239.6	181814_at
WBGene00019505	K07H8.3	181918_at
WBGene00006574	tin-13	182021_at
WBGene00015313	C01G8.6	182043_s_at
WBGene00019017	F57F4.4	182088_s_at
WBGene00014183	ZK1025.3	182104_at
WBGene00022751	ZK484.5	182193_at
WBGene00021095	mlt-8	182327_at
WBGene00022517	ZC123.1	182346_at
WBGene00012540	Y37A1B.7	182386_s_at
WBGene00011035	R05D11.7	182400_at
WBGene00012452	Y17D7B.4	182439_at
WBGene00016474	C36C5.5	182575_at
WBGene00016805	C50D2.1	182629_at
WBGene00020391	cct-7	182656_at
WBGene00019357	cpg-8	182696_s_at
WBGene00018677	F52C9.7	182792_at
WBGene00019824	R02D3.7	182842_s_at
WBGene00022694	ZK328.4	182907_s_at
WBGene00018965	F56D2.3	182923_at
WBGene00008768	F13G3.10	182982_at
WBGene00017307	F09F9.2	183159_at
WBGene00001233	eif-3.K	183172_s_at
WBGene00019727	M02D8.1	183201_at

WBGene00022679	ZK180.5	183237_s_at
WBGene00010425	H37A05.1	183240_s_at
WBGene00010468	K01D12.9	183379_at
WBGene00017349	F10E7.8	183396_s_at
WBGene00019323	tag-203	183546_s_at
WBGene00004174	pqn-95	183577_at
WBGene00010296	F59A1.10	183744_at
WBGene00016422	noah-1	183775_at
WBGene00020796	T25D3.2	183795_s_at
WBGene00021097	cdc-37	183837_s_at
WBGene00018393	F43E2.5	183902_at
WBGene00014187	ZK1025.7	183907_at
WBGene00018969	F56D3.1	183941_s_at
WBGene00016790	C49H3.3	183984_at
WBGene00002068	ify-1	184075_s_at
WBGene00017317	F09G2.9	184089_s_at
WBGene00014182	ZK1025.2	184102_s_at
WBGene00011487	T05E12.6	184116_s_at
WBGene00016440	C35D10.2	184202_at
WBGene00020915	nol-5	184243_s_at
WBGene00012522	Y32B12B.2	184244_at
WBGene00001971	hmg-1.1	184409_s_at
WBGene00015949	C18A11.3	184431_at
WBGene00013349	Y59A8B.12	184432_at
WBGene00018064	F35F11.1	184648_at
WBGene00020530	T15B12.1	184664_at
WBGene00019247	H27M09.3	184667_at
WBGene00006956	wrt-10	184676_s_at
WBGene00017641	csr-1	184755_at
WBGene00021533	Y42G9A.3	184843_at
WBGene00017993	F32E10.6	184965_s_at
WBGene00017605	F19F10.9	185007_s_at
WBGene00015075	B0238.11	185022_at
WBGene00012996	Y48C3A.16	185131_at
WBGene00016057	C24D10.6	185237_s_at
WBGene00021004	W03F9.10	185268_at
WBGene00019839	R02F11.1	185334_s_at
WBGene00018811	pmt-2	185350_at
WBGene00018045	F35D11.4	185416_at
WBGene00017998	F33D4.6	185424_s_at
WBGene00001724	grl-15	185468_at
WBGene00018340	F42A10.3	185506_at
WBGene00018046	F35D11.5	185524_at
WBGene00021785	Y51H7C.7	185584_at
WBGene00019236	H23N18.5	185593_at
WBGene00006587	tnt-2	185643_s_at
WBGene00001703	grd-14	185646_at
WBGene00018762	F53E10.6	185695_s_at
WBGene00013606	cand-1	185737_at

WBGene00017998	F33D4.6	185764_s_at
WBGene00004104	pqn-13	185795_at
WBGene00022194	Y71H2B.4	185811_at
WBGene00019520	K08B12.1	185835_at
WBGene00004754	sec-23	185913_at
WBGene00020014	R11G11.6	185916_at
WBGene00019272	H42K12.3	185972_at
WBGene00000549	cls-2	186020_s_at
WBGene00019003	F57B10.5	186058_at
WBGene00019914	clec-150	186278_at
WBGene00006924	vig-1	186328_at
WBGene00018380	F43C11.3	186337_at
WBGene00004919	snr-6	186350_at
WBGene00004920	snr-7	186387_at
WBGene00013219	Y54G11A.11	186483_at
WBGene00017490	F15E11.1	186492_s_at
WBGene00016449	C35D10.13	186540_at
WBGene00010227	F58A4.2	186566_s_at
WBGene00009778	F46C5.1	186660_s_at
WBGene00021934	cct-8	186663_at
WBGene00022857	ZK1127.12	186731_at
WBGene00019168	H06I04.3	186870_s_at
WBGene00012483	Y18D10A.16	186889_at
WBGene00007022	mdt-22	186907_at
WBGene00012484	car-1	186915_at
WBGene00001725	grl-16	186927_s_at
WBGene00013958	ZK265.6	186929_at
WBGene00000941	ddp-1	186934_s_at
WBGene00021904	Y55B1AL.2	187018_at
WBGene00013263	Y57A10A.23	187073_at
WBGene00006529	tba-2	187081_s_at
WBGene00008398	D2005.3	187087_at
WBGene00019285	cbn-1	187124_s_at
WBGene00001371	exl-1	187188_s_at
WBGene00020894	T28D9.1	187211_at
WBGene00021959	Y57E12AL.6	187271_at
WBGene00015205	B0495.6	187349_at
WBGene00021921	Y55F3AM.3	187389_s_at
WBGene00001934	his-60	187410_at
WBGene00007625	C16C10.4	187439_at
WBGene00019607	K10B2.4	187451_at
WBGene00014938	Y62E10A.11	187471_at
WBGene00018132	F37A4.2	187472_at
WBGene00019630	K10D2.4	187482_at
WBGene00015207	B0495.8	187511_s_at
WBGene00014224	ZK1098.7	187526_at
WBGene00022579	ZC262.2	187564_at
WBGene00017830	rpb-8	187604_s_at
WBGene00022765	ZK546.14	187657_s_at

WBGene00004888	smo-1	187669_at
WBGene00000479	cgh-1	187802_at
WBGene00010908	M88.5	187934_at
WBGene00003992	pgl-1	187974_s_at
WBGene00004464	rpn-8	187980_at
WBGene00009237	F28H7.3	187996_s_at
WBGene00006984	zig-7	188008_s_at
WBGene00006948	wrt-2	188061_s_at
WBGene00004781	set-1	188087_at
WBGene00001113	dur-1	188104_s_at
WBGene00004244	puf-8	188144_s_at
WBGene00001977	hmg-12	188169_at
WBGene00001072	dpy-10	188200_at
WBGene00004875	smd-1	188304_at
WBGene00001386	far-2	188325_at
WBGene00000705	col-131	188331_s_at
WBGene00015047	B0218.1	188358_at
WBGene00003057	lon-3	188368_at
WBGene00000726	col-153	188375_at
WBGene00000685	col-111	188461_s_at
WBGene00015752	C14B9.2	188474_s_at
WBGene00000381	cct-6	188482_s_at
WBGene00000039	acn-1	188527_s_at
WBGene00002083	inf-1	188535_s_at
WBGene00001000	dim-1	188558_s_at
WBGene00001854	hil-3	188654_at
WBGene00003795	npp-9	188655_s_at
WBGene00021088	W08E12.7	188669_at
WBGene00001928	his-54	188677_s_at
WBGene00011038	R05H5.3	188696_at
WBGene00012277	W05B10.2	188709_s_at
WBGene00001855	hil-4	188717_at
WBGene00000896	dad-1	188737_at
WBGene00001856	hil-5	188740_at
WBGene00011559	T07C4.1	188749_s_at
WBGene00001031	dnj-13	188802_at
WBGene00003214	mel-32	188803_s_at
WBGene00011182	R09H10.5	188852_s_at
WBGene00000121	aly-2	188872_at
WBGene00008921	F17C11.10	188876_s_at
WBGene00007329	C05C10.2	188899_s_at
WBGene00001047	dnj-29	188943_s_at
WBGene00004736	sca-1	188971_s_at
WBGene00001423	fib-1	189006_s_at
WBGene00008053	cdc-48.2	189021_at
WBGene00000228	atn-1	189086_s_at
WBGene00010912	M106.4	189093_at
WBGene00009706	F44G3.2	189178_at
WBGene00013983	ZK512.2	189211_s_at

WBGene00008810	cyp-13A12	189219_s_at
WBGene00002070	ile-1	189324_s_at
WBGene00019246	rpb-5	189330_at
WBGene00011408	T04A8.6	189360_s_at
WBGene00011128	R07H5.8	189371_s_at
WBGene00003920	par-5	189382_s_at
WBGene00019470	cyp-35B3	189398_at
WBGene00003081	lsm-7	189411_at
WBGene00000512	ckb-2	189460_at
WBGene00017984	F32D1.5	189508_s_at
WBGene00011758	T13H5.4	189513_s_at
WBGene00006724	ubh-4	189514_at
WBGene00020283	T06A4.3	189521_s_at
WBGene00020190	T03F1.8	189532_at
WBGene00010204	F57F5.1	189544_at
WBGene00000067	act-5	189551_s_at
WBGene00020683	T22D1.4	189552_at
WBGene00010405	H19N07.1	189573_s_at
WBGene00004700	rsp-3	189582_s_at
WBGene00006536	tbb-1	189586_s_at
WBGene00021002	W03F9.4	189587_s_at
WBGene00007107	pfd-4	189618_at
WBGene00001333	erm-1	189677_s_at
WBGene00020340	T08B1.1	189686_at
WBGene00019401	nuo-4	189698_s_at
WBGene00004387	rnp-4	189726_at
WBGene00004302	ran-1	189729_s_at
WBGene00019644	cpt-4	189736_s_at
WBGene00013575	Y76A2B.3	189748_s_at
WBGene00011272	R53.2	189752_s_at
WBGene00020909	W01A11.1	189755_at
WBGene00002007	hsp-3	189828_s_at
WBGene00001819	haf-9	189882_at
WBGene00003533	nas-14	189899_at
WBGene00019947	htz-1	189914_s_at
WBGene00004459	rpn-2	189994_at
WBGene00001071	dpy-9	190013_at
WBGene00003553	nas-37	190032_s_at
WBGene00002219	klp-7	190047_s_at
WBGene00005017	sqt-2	190091_at
WBGene00020788	T25B6.2	190121_s_at
WBGene00000618	col-41	190134_s_at
WBGene00006608	tre-2	190186_s_at
WBGene00004007	pgp-13	190218_at
WBGene00003950	pbs-4	190229_at
WBGene00004701	rsp-4	190239_s_at
WBGene00011391	T03D8.2	190280_at
WBGene00001884	his-10	190307_s_at
WBGene00009772	ztf-7	190314_at

WBGene00000206	asb-1	190337_s_at
WBGene00011880	T21B6.3	190368_s_at
WBGene00003816	nrs-2	190413_at
WBGene00002238	krs-1	190443_s_at
WBGene00001070	dpy-8	190446_s_at
WBGene00001635	gly-10	190454_at
WBGene00006764	unc-27	190469_at
WBGene00012964	Y48A6B.3	190470_at
WBGene00011284	ttr-27	190473_at
WBGene00018362	F42G9.1	190487_at
WBGene00020808	T25F10.6	190546_s_at
WBGene00011285	ttr-28	190564_at
WBGene00020757	ucr-2.3	190569_at
WBGene00010629	K07C5.6	190576_s_at
WBGene00020022	R12C12.1	190616_at
WBGene00003934	pat-10	190659_s_at
WBGene00001598	glh-1	190711_at
WBGene00017332	ugt-37	190719_at
WBGene00010785	top-2	190785_s_at
WBGene00001337	ers-2	190790_at
WBGene00002064	iff-1	190791_at
WBGene00010317	F59B8.2	190796_s_at
WBGene00001909	his-35	190805_at
WBGene00020812	T25G12.5	190819_s_at
WBGene00000107	alh-1	190851_at
WBGene00022119	Y71F9AL.17	190887_s_at
WBGene00010573	K04H4.2	190901_s_at
WBGene00010790	sodh-1	190978_at
WBGene00011850	T20B3.1	190999_at
WBGene00001073	dpy-11	191000_s_at
WBGene00019184	H10E21.4	191001_s_at
WBGene00020366	T08G2.3	191041_s_at
WBGene00011412	T04A8.11	191048_at
WBGene00013077	ttr-24	191098_at
WBGene00000209	asg-1	191143_at
WBGene00000209	asg-1	191144_s_at
WBGene00006947	wrt-1	191180_at
WBGene00009650	F43D2.1	191203_at
WBGene00004044	plk-3	191208_s_at
WBGene00017937	F30H5.3	191217_s_at
WBGene00001239	elo-1	191269_at
WBGene00003958	pcp-3	191274_s_at
WBGene00003803	npp-17	191350_s_at
WBGene00021292	Y25C1A.5	191428_s_at
WBGene00001774	gst-26	191431_at
WBGene00018271	F41C3.5	191462_at
WBGene00009142	F26A3.4	191521_s_at
WBGene00008682	lex-1	191580_s_at
WBGene00017982	F32D1.2	191648_at

WBGene00004679	rrt-1	191704_s_at
WBGene00014165	puf-12	191768_at
WBGene00001497	frs-1	191771_s_at
WBGene00009973	F53C11.3	191783_at
WBGene00003777	nmy-2	191813_s_at
WBGene00010627	K07C5.4	191820_s_at
WBGene00010678	K08F4.3	191833_s_at
WBGene00017125	E04F6.5	191847_s_at
WBGene00006789	unc-54	191926_s_at
WBGene00007355	rpb-6	191936_at
WBGene00002008	hsp-4	192010_s_at
WBGene00019162	H06H21.3	192041_s_at
WBGene00001999	hrp-1	192043_s_at
WBGene00009319	F32B6.2	192078_at
WBGene00017438	F13H8.5	192089_s_at
WBGene00003924	pas-3	192103_s_at
WBGene00006626	tsn-1	192151_at
WBGene00000275	bub-1	192167_at
WBGene00004703	rsp-6	192183_s_at
WBGene00015538	C06E7.1	192191_s_at
WBGene00006510	tag-165	192200_at
WBGene00018997	F57B9.3	192240_s_at
WBGene00010230	F58A4.9	192261_s_at
WBGene00010896	M28.5	192276_at
WBGene00020324	T07F12.4	192308_s_at
WBGene00001397	fat-5	192357_at
WBGene00007836	C31C9.2	192474_s_at
WBGene00007129	B0272.3	192491_at
WBGene00008205	sams-1	192492_at
WBGene00008205	sams-1	192493_s_at
WBGene00017123	maoc-1	192499_at
WBGene00011938	T22H6.2	192504_at
WBGene00009002	hint-1	192514_at
WBGene00015540	C06E7.3	192547_s_at
WBGene00001399	fat-7	192578_at
WBGene00004305	ran-4	192589_at
WBGene00004766	sel-9	192625_s_at
WBGene00009211	F28D1.1	192639_s_at
WBGene00003511	mxl-3	192645_at
WBGene00005016	sqt-1	192674_s_at
WBGene00004397	rol-6	192682_at
WBGene00002073	ima-2	192711_at
WBGene00017991	clec-180	192712_at
WBGene00006416	tag-32	192822_s_at
WBGene00004320	rbx-1	192856_at
WBGene00020114	pde-4	192893_at
WBGene00006528	tba-1	192922_s_at
WBGene00003844	odc-1	192924_s_at
WBGene00003925	pas-4	192934_s_at

WBGene00003962	pdi-1	192946_s_at
WBGene00004398	rol-8	192967_at
WBGene00004398	rol-8	192968_s_at
WBGene00002258	lbp-6	192988_at
WBGene00016844	C50F7.4	192995_at
WBGene00004915	snr-2	193023_at
WBGene00009069	F23A7.4	193033_at
WBGene00004468	rpn-12	193048_at
WBGene00000996	dif-1	193110_at
WBGene00004916	snr-3	193119_s_at
WBGene00002254	lbp-2	193132_at
WBGene00010738	K10D3.4	193156_s_at
WBGene00010478	K01G5.5	193161_at
WBGene00004358	rhr-1	193165_at
WBGene00006787	unc-52	193208_s_at
WBGene00013984	ZK512.4	193232_at
WBGene00000967	dhs-3	193240_s_at
WBGene00003159	mcm-7	193260_s_at
WBGene00017990	F32E10.2	193306_at
WBGene00002057	ifd-1	193321_at
WBGene00018737	F53B1.4	193327_at
WBGene00006366	sym-1	193344_at
WBGene00004422	rpl-11.1	193434_s_at
WBGene00008449	E01G6.1	193477_s_at
WBGene00001973	hmg-3	193484_s_at
WBGene00011638	T09A5.11	193500_s_at
WBGene00006757	unc-18	193527_at
WBGene00006921	vha-12	193564_s_at
WBGene00007245	C01G12.5	193598_at
WBGene00007455	ugt-22	193604_at
WBGene00003079	lsm-5	193620_at
WBGene00019322	K02F2.2	193627_at
WBGene00007135	B0285.1	193628_at
WBGene00010560	iftb-1	193644_s_at
WBGene00013311	Y57G11C.15	193652_s_at
WBGene00001430	fkb-5	193656_s_at
WBGene00001331	erd-2	193692_s_at
WBGene00022046	Y66H1A.4	193704_at
WBGene00000122	aly-3	193709_at
WBGene00020269	T05H4.6a	193710_at
WBGene00015781	C14F11.6	193712_s_at
WBGene00000376	ccr-4	193716_s_at
WBGene00003163	mdl-1	193723_s_at
WBGene00000877	cyn-1	193725_at
WBGene00002196	kin-10	193737_s_at
WBGene00003964	pdi-3	193755_s_at
WBGene00004391	rnr-1	193772_s_at
WBGene00004808	skr-2	193775_at
WBGene00004914	snr-1	193791_s_at

WBGene00007565	clec-48	193801_s_at
WBGene00001595	gld-1	193805_at
WBGene00011511	T05H10.7	193820_s_at
WBGene00012253	clec-50	193836_s_at
WBGene00001051	cks-1	193886_at
WBGene00000040	aco-1	193891_at
WBGene00010988	R03D7.1	193894_at
WBGene00000070	acy-3	193920_at
WBGene00000405	cdk-1	193939_at
WBGene00001303	emo-1	193944_at
WBGene00007352	cdc-48.1	193984_s_at
WBGene00013025	vha-13	193987_s_at
WBGene00004705	rsp-8	194005_s_at
WBGene00012019	dkf-2	194083_s_at
WBGene00012186	mlt-11	194086_at
WBGene00009740	F45H10.3	194108_s_at
WBGene00013668	Y105E8A.3	194158_s_at
WBGene00020894	T28D9.1	194160_s_at
WBGene00022025	Y65B4A.1	194172_s_at
WBGene00012186	mlt-11	194187_x_at
WBGene00012261	lpr-3	194203_x_at
WBGene00018533	F47B7.2	194210_x_at
WBGene00001830	hcp-2	194214_x_at
WBGene00021427	Y38F2AR.9	194222_x_at
WBGene00044357	nspc-6	194223_x_at
WBGene00008605	mlt-9	194238_x_at
WBGene00004698	rsp-1	194244_x_at
WBGene00009634	nspc-9	194245_x_at
WBGene00009640	nspc-10	194245_x_at
WBGene00017691	ilys-5	194255_x_at
