# Supplementary material for: GEI-8, a Homologue of Vertebrate Nuclear Receptor Corepressor NCoR/SMRT, Regulates Gonad Development and Neuronal Functions in Caenorhabditis elegans
Source: PLoS One. 2013 Mar 6;8(3):e58462. doi: 10.1371/journal.pone.0058462 (PMC3590189; doi:10.1371/journal.pone.0058462)
Supplement: Table S2 — List of genes with increased expression in gei-8(ok1671) homozygous mutants. (PDF) [file pone.0058462.s005.pdf]

Table S2. List of genes with increased expression in *gei-8(ok1671)* homozygous mutants.

Gene WB ID	Gene Public Name	Oligo Set
WBGene00006928	vit-4	171723_x_at
WBGene00011737	T12G3.1	171737_x_at
WBGene00011474	T05D4.1	171820_x_at
WBGene00000783	cpr-3	172069_x_at
WBGene00006927	vit-3	172133_x_at
WBGene00006928	vit-4	172134_x_at
WBGene00019207	H17B01.1	172183_at
WBGene00011733	T12D8.5	172193_at
WBGene00007992	fipr-24	172241_x_at
WBGene00003769	nlp-31	172343_x_at
WBGene00003767	nlp-29	172375_x_at
WBGene00003768	nlp-30	172386_x_at
WBGene00003765	nlp-27	172388_x_at
WBGene00003766	nlp-28	172427_x_at
WBGene00000556	cnc-2	172437_x_at
WBGene00017232	F07G11.7	172508_x_at
WBGene00012813	Y43F8B.2	172578_x_at
WBGene00017396	F12A10.7	172686_x_at
WBGene00006929	vit-5	172696_x_at
WBGene00000599	col-10	172723_x_at
WBGene00003473	mtl-1	172744_at
WBGene00002020	hsp-16.49	172901_x_at
WBGene00008741	F13D12.6	172958_s_at
WBGene00000219	asp-6	173036_s_at
WBGene00020311	T07D3.9	173073_s_at
WBGene00008741	F13D12.6	173089_at
WBGene00009396	clec-65	173090_s_at
WBGene00021872	clec-85	173144_at
WBGene00003702	nhr-112	173159_s_at
WBGene00022199	Y71H10A.1	173265_at
WBGene00012185	W01F3.2	173274_s_at
WBGene00001564	gei-7	173301_s_at
WBGene00011305	R186.1	173404_s_at
WBGene00006930	vit-6	173411_s_at
WBGene00003891	osm-11	173523_s_at
WBGene00015163	B0361.9	173537_at
WBGene00001685	gpd-3	173608_s_at
WBGene00011231	R11A5.3	173687_s_at
WBGene00015132	B0303.14	173796_at
WBGene00008780	F14B4.2	173814_at
WBGene00000065	act-3	173992_at
WBGene00017854	F27C1.4	174134_at

WBGene00009243	F29C6.1	174420_at
WBGene00002263	lea-1	174552_s_at
WBGene00009221	acs-2	174675_at
WBGene00010659	K08D8.5	175126_s_at
WBGene00021219	Y19D10A.4	175253_s_at
WBGene00007533	C12C8.2	175512_s_at
WBGene00021219	Y19D10A.4	175532_at
WBGene00016048	C24B9.3	175591_s_at
WBGene00003166	mec-2	175614_at
WBGene00020135	T01B6.3	176009_at
WBGene00021518	Y41D4B.16	176044_at
WBGene00005501	srh-297	176127_at
WBGene00021977	Y58A7A.3	176141_s_at
WBGene00021979	Y58A7A.5	176209_at
WBGene00021219	Y19D10A.4	176338_at
WBGene00018028	F35B3.1	176689_at
WBGene00016658	C45B2.1	176692_s_at
WBGene00016662	C45B2.8	176692_s_at
WBGene00021491	Y40B10A.6	176851_at
WBGene00000066	act-4	176923_s_at
WBGene00021945	Y55F3BR.8	177095_at
WBGene00044757	Y55F3BR.11	177095_at
WBGene00021396	Y38C1AA.5	177112_at
WBGene00019207	H17B01.1	177128_at
WBGene00019780	M60.4	177279_at
WBGene00010935	M163.1	177298_at
WBGene00011293	R102.6	177339_at
WBGene00019779	M60.2	177375_at
WBGene00007807	C29F3.7	177487_at
WBGene00012034	T26C5.4	177598_at
WBGene00010216	F57G8.7	177613_at
WBGene00009485	F36G9.3	177718_at
WBGene00012408	Y7A5A.2	177799_at
WBGene00011706	T11B7.1	177991_at
WBGene00009623	F41E7.7	178094_at
WBGene00012044	T26E4.2	178235_at
WBGene00011979	T24B8.5	178297_at
WBGene00007989	fipr-22	178571_s_at
WBGene00008681	scrm-4	178635_at
WBGene00002107	ins-24	178669_s_at
WBGene00009803	F47B8.2	178834_at
WBGene00012140	T28F4.5	178922_at
WBGene00010041	F54C9.3	179078_s_at
WBGene00008217	C50B6.1	179079_at
WBGene00008816	F14F8.8	179137_at
WBGene00008157	C47F8.1	179143_at
WBGene00009787	F46F2.3	179187_s_at
WBGene00010019	F54B8.4	179214_at
WBGene00008862	F15D4.5	179266_at

WBGene00007365	C06B3.6	179272_at
WBGene00014053	ZK669.3	179285_at
WBGene00009199	F28B1.5	179364_at
WBGene00012398	Y6E2A.4	179418_at
WBGene00012398	Y6E2A.4	179452_at
WBGene00011292	R102.5	179489_s_at
WBGene00009805	F47B8.4	179492_at
WBGene00013962	ZK287.1	179539_at
WBGene00010001	F53F8.4	179553_at
WBGene00007689	C18E9.9	179607_at
WBGene00008404	D2013.6	179636_at
WBGene00010658	K08D8.4	179650_at
WBGene00008975	F20D1.3	179692_at
WBGene00009429	F35E12.5	179695_at
WBGene00008739	F13D12.3	179714_at
WBGene00012783	Y43C5A.3	179727_at
WBGene00013065	Y51A2B.1	179786_at
WBGene00021048	W05H9.1	179806_at
WBGene00018348	F42C5.6	179808_at
WBGene00012940	Y47D3B.3	179881_at
WBGene00012941	Y47D3B.4	179881_at
WBGene00020311	T07D3.9	179891_s_at
WBGene00017207	fbxb-6	180197_at
WBGene00017485	F15E6.4	180398_s_at
WBGene00016061	C24G6.6	180541_s_at
WBGene00019453	K06H6.5	180645_at
WBGene00010135	F55H12.4	180707_at
WBGene00006135	str-74	180740_at
WBGene00015573	C07G1.7	180773_at
WBGene00019563	K09C6.9	180786_at
WBGene00011190	R10D12.9	180810_at
WBGene00011191	R10D12.10	180810_at
WBGene00018643	F49F1.1	180925_at
WBGene00018646	F49F1.6	180973_at
WBGene00018647	F49F1.7	181132_s_at
WBGene00020596	oga-1	181147_s_at
WBGene00017117	fbxb-80	181198_at
WBGene00017488	dct-7	181270_at
WBGene00021011	W03G1.5	181281_at
WBGene00012980	Y48B6A.6	181498_s_at
WBGene00018608	F48E3.9	181649_at
WBGene00018602	F48D6.4	181694_at
WBGene00013089	Y51B9A.7	181764_at
WBGene00007231	C01G10.4	182405_at
WBGene00044658	C01G10.17	182405_at
WBGene00017303	F09F7.6	182470_at
WBGene00015828	math-14	182492_at
WBGene00007833	C31A11.5	182733_at
WBGene00020760	T24C4.4	182815_at

WBGene00022757	ZK488.5	182892_at
WBGene00004157	pqn-75	182930_at
WBGene00018165	F38A5.8	182939_at
WBGene00010124	F55G11.4	182970_at
WBGene00016048	C24B9.3	183010_s_at
WBGene00019090	F59A7.2	183014_at
WBGene00019921	R07C3.5	183027_at
WBGene00003091	lys-2	183028_s_at
WBGene00020725	T23C6.3	183310_s_at
WBGene00010128	F55G11.8	183378_at
WBGene00016923	C54F6.5	183441_at
WBGene00010746	K10D11.2	183442_at
WBGene00044073	tag-244	183555_at
WBGene00005684	sru-21	183556_at
WBGene00015756	C14C6.2	183567_s_at
WBGene00017964	F31F7.1	183621_at
WBGene00010125	dod-22	183624_at
WBGene00022731	ZK402.5	183757_at
WBGene00015574	C07G3.2	183876_at
WBGene00020445	T12B3.2	183900_at
WBGene00011428	T04C12.3	183970_at
WBGene00015231	B0511.5	183991_at
WBGene00021106	W09B7.1	184014_s_at
WBGene00021107	W09B7.2	184103_s_at
WBGene00016419	tyr-4	184158_at
WBGene00010262	F58E10.7	184175_at
WBGene00000558	cnc-4	184191_at
WBGene00017858	F27C1.11	184239_at
WBGene00019660	K11H12.4	184313_s_at
WBGene00017726	F22H10.2	184400_at
WBGene00019664	K11H12.8	184428_at
WBGene00019664	K11H12.8	184429_s_at
WBGene00016027	C23H5.8	184500_s_at
WBGene00015933	C17H12.8	184533_at
WBGene00015933	C17H12.8	184534_s_at
WBGene00019909	R06A10.1	184615_at
WBGene00018439	fbxa-184	184703_at
WBGene00007873	dod-21	184707_s_at
WBGene00021183	Y9C9A.16	184710_at
WBGene00018987	F56F10.4	184753_at
WBGene00017390	F12A10.1	184868_at
WBGene00017396	F12A10.7	184868_at
WBGene00019580	K09E10.2	184950_at
WBGene00015076	B0238.12	185026_at
WBGene00009434	F35E12.10	185153_s_at
WBGene00021714	cyh-1	185221_at
WBGene00010473	cdr-5	185275_at
WBGene00003759	nlp-21	185393_s_at
WBGene00003096	lys-7	185557_s_at

WBGene00016027	C23H5.8	185619_s_at
WBGene00016097	C25E10.8	185690_at
WBGene00019021	F57H12.6	186012_s_at
WBGene00013633	Y105C5A.8	186052_at
WBGene00019968	R08F11.4	186072_s_at
WBGene00019541	K08F11.1	186083_at
WBGene00019830	R02E12.6	186182_s_at
WBGene00018650	F49F1.10	186243_at
WBGene00013629	srz-95	186509_at
WBGene00017497	F15E11.11	186523_s_at
WBGene00013986	ZK512.7	186714_s_at
WBGene00012591	Y38E10A.13	186726_at
WBGene00018460	F45D11.15	186801_s_at
WBGene00009192	F27E5.1	186832_at
WBGene00015935	C17H12.10	187029_at
WBGene00021141	clec-134	187076_at
WBGene00010320	F59B10.4	187183_s_at
WBGene00017698	F22B7.9	187282_at
WBGene00008741	F13D12.6	187323_at
WBGene00015163	B0361.9	187438_s_at
WBGene00022439	Y110A2AL.2	187441_at
WBGene00022725	ZK381.2	187460_at
WBGene00015265	B0563.8	187504_at
WBGene00022674	ZK177.9	187540_at
WBGene00020737	T23F2.4	187571_at
WBGene00002263	lea-1	187999_at
WBGene00001581	gfi-1	188046_s_at
WBGene00001113	dur-1	188100_s_at
WBGene00000633	col-56	188191_at
WBGene00002016	hsp-16.2	188282_at
WBGene00002088	ins-5	188319_at
WBGene00004394	rol-1	188337_at
WBGene00000673	col-98	188356_at
WBGene00002018	hsp-16.41	188444_at
WBGene00000715	col-142	188500_at
WBGene00014206	nit-1	188607_at
WBGene00000656	col-80	188622_at
WBGene00001168	eft-3	188725_s_at
WBGene00016316	C32D5.8	188744_s_at
WBGene00006628	tsp-2	188927_at
WBGene00019232	ugt-13	188934_at
WBGene00017121	cyc-2.1	188940_at
WBGene00010759	K10H10.2	188978_s_at
WBGene00001229	eif-3.F	189010_s_at
WBGene00007507	C10C5.3	189069_at
WBGene00002015	hsp-16.1	189087_s_at
WBGene00005228	srh-2	189126_s_at
WBGene00008451	E01G6.3	189234_at
WBGene00016147	cyp-32A1	189264_s_at

WBGene00008850	F15B9.6	189419_at
WBGene00000748	col-175	189482_s_at
WBGene00019967	cyp-33C8	189519_at
WBGene00000282	cah-4	189628_at
WBGene00000749	col-176	189629_at
WBGene00008591	F08H9.3	189643_at
WBGene00006539	tbb-6	189718_at
WBGene00019890	R05F9.6	189767_at
WBGene00019207	H17B01.1	189933_s_at
WBGene00000615	col-38	189968_s_at
WBGene00018986	F56F10.3	190361_s_at
WBGene00001500	ftn-1	190427_s_at
WBGene00014173	ZK970.7	190585_at
WBGene00007605	C15C8.3	190619_at
WBGene00017970	F32A5.4	190628_at
WBGene00000287	cal-3	190642_s_at
WBGene00017565	F18E3.7	190646_s_at
WBGene00006930	vit-6	190649_at
WBGene00019584	set-12	190725_at
WBGene00010493	meg-2	190730_at
WBGene00007304	ttr-39	190896_at
WBGene00012383	ttr-17	190906_at
WBGene00001762	gst-14	190929_at
WBGene00000115	alh-9	190995_at
WBGene00001803	lite-1	191026_s_at
WBGene00006627	tsp-1	191038_at
WBGene00005477	srh-271	191265_at
WBGene00006926	vit-2	191323_at
WBGene00005551	sri-39	191340_at
WBGene00005142	srd-65	191387_at
WBGene00005236	srh-10	191493_at
WBGene00000412	cdr-1	191611_at
WBGene00010539	ttr-2	191676_at
WBGene00012530	Y32F6A.5	191775_s_at
WBGene00010470	cdr-4	191871_s_at
WBGene00012144	T28H10.3	192181_at
WBGene00009221	acs-2	192195_at
WBGene00002026	hsp-70	192266_s_at
WBGene00002012	hsp-12.3	192338_at
WBGene00001752	gst-4	192407_at
WBGene00023407	cex-1	192450_at
WBGene00007947	C35A5.3	192528_at
WBGene00009396	clec-65	192559_s_at
WBGene00021043	W05G11.6	192581_s_at
WBGene00004932	sod-3	192617_at
WBGene00004735	sbp-1	192735_s_at
WBGene00011474	T05D4.1	192888_s_at
WBGene00002262	ldh-1	192972_at
WBGene00005942	srx-51	193367_at

WBGene00003655	nhr-65	193567_at
WBGene00009153	F26D2.15	193573_at
WBGene00003651	nhr-61	193603_s_at
WBGene00003602	nhr-3	193679_s_at
WBGene00009397	clec-66	194067_at
WBGene00001564	gei-7	194142_x_at
WBGene00009963	fipr-26	194157_s_at
WBGene00001684	gpd-2	194177_x_at
WBGene00006927	vit-3	194239_x_at
