# Supplementary material for: GEI-8, a Homologue of Vertebrate Nuclear Receptor Corepressor NCoR/SMRT, Regulates Gonad Development and Neuronal Functions in Caenorhabditis elegans
Source: PLoS One. 2013 Mar 6;8(3):e58462. doi: 10.1371/journal.pone.0058462 (PMC3590189; doi:10.1371/journal.pone.0058462)
Supplement: Table S3 — Primers used in the study. (PDF) [file pone.0058462.s006.pdf]

Table S3. Primers used in the study.

107: ATG AGT CTG ATG ATG GTG AGG AGG AC

307: CTT GAG CCT TCA AAT GTG CCT GCT G

01/021: CTG CAG TGG CTC TTT GTA ACG TTC

01/042: TCT TCA CCG CGG ATT CGT TCA GC

05/153: CGA CAA CCC GAA CAA ACA ACT CG

4938: GGA TCC ACT TTT CAG TGG TCG GGC

5056: AAC TGC AGT CTT TAA CTC CGA GTT TGT TTC CC

5060: CGG GGA TCC AGA TCA TCT TGA AAG GCT TTG AAC T

6158: GGG GTA CCA CTC ATC AC GAC ACT TGA TTG GG

6168: CGG GAT CCC ACA AGT GGA AGG AGG GAA TAG GA

6173: CGC TTG ATG TTG TAG GTG TAT TCA CC

6174: AAC TGC AGC AGG CCT CAT CTT GCG C

6200: CGG GAT CCA TCG GTT GAA GAG CTT GCA TAC

6242: CTT GAA TAT CAT ATG TGT TTG CGT G

6243: TAT TTG GTT GTT TAA TCG GTG ATT C

6228: CAG CAG CAG GCC TCA TCT TGC GCG

6229: CTG TAC TCT CTT CAC TAT TGA CGC

6230: AGT CGA CCT GCA GGC ATG CAA GCT ATC TTC ACC GCG GAT TCG TTC AGC

6232: AGC TTG CAT GCC TGC AGG TCG ACT

6233: AAG GGC CCG TAC GGC CGA CTA GTA GG

6234: GGA AAC AGT TAT GTT TGG TAT ATT GGG

7144: CTG GAC TAG AAA ATA ACT TCT GAA GTG CTT C

7149: ATG GAA AGC TTC AAA GAG CTC GCC

7501: AGC ACT ATC AAG AAG TTA TGA ACT

7502: TGT TCA TAC AAT CTC CAA CAC CAA

SL1: GGT TTA ATT ACC CAA GTT TGA G

7605: AGT AGC TCG GGA TAA GGA AAT CG

7606: TAA ATC CAG TTT TAT CGA CAA ATG C

7749: GGC GTC AAC TCC TTC TAT GGA TCA

7750: TTA TTC ATC CAA TTC CTT CAC TAT T

7751: TCT CGG CCA GAT GAA ACA ACG TTA TGA G

7752: TTA CAT CTG AAT TAT TTG TGT TTG CAG GT

7753: ACT TGC TGA GAG GAT GAA TGA AGT TTT C

7754: CAT CTA TTC ATC ATC CGA TAA GTC

10/44: CGG GAT CCA TGA TTC AAT CCA GTT CTT CAA TAA

10/45: CGG AAT TCT TAC GAG TCC ATT ATA TCT TCT ATA ACT
